# The transcriptional correlates of divergent electric organ discharges in *Paramormyrops* electric fish

**DOI:** 10.1186/s12862-019-1572-3

**Published:** 2020-01-09

**Authors:** Mauricio Losilla, David Michael Luecke, Jason R. Gallant

**Affiliations:** 1 Department of Integrative Biology, Michigan State University, East Lansing, MI 48824 USA; 2 Graduate Program in Ecology, Evolutionary Biology and Behavior, Michigan State University, East Lansing, MI 48824 USA

## Abstract

**Background:**

Understanding the genomic basis of phenotypic diversity can be greatly facilitated by examining adaptive radiations with hypervariable traits. In this study, we focus on a rapidly diverged species group of mormyrid electric fish in the genus *Paramormyrops*, which are characterized by extensive phenotypic variation in electric organ discharges (EODs). The main components of EOD diversity are waveform duration, complexity and polarity. Using an RNA-sequencing based approach, we sought to identify gene expression correlates for each of these EOD waveform features by comparing 11 specimens of *Paramormyrops* that exhibit variation in these features.

**Results:**

Patterns of gene expression among *Paramormyrops* are highly correlated, and 3274 genes (16%) were differentially expressed. Using our most restrictive criteria, we detected 145–183 differentially expressed genes correlated with each EOD feature, with little overlap between them. The predicted functions of several of these genes are related to extracellular matrix, cation homeostasis, lipid metabolism, and cytoskeletal and sarcomeric proteins. These genes are of significant interest given the known morphological differences between electric organs that underlie differences in the EOD waveform features studied.

**Conclusions:**

In this study, we identified plausible candidate genes that may contribute to phenotypic differences in EOD waveforms among a rapidly diverged group of mormyrid electric fish. These genes may be important targets of selection in the evolution of species-specific differences in mate-recognition signals.

## Background

Understanding the genomic basis of phenotypic diversity is a major goal of evolutionary biology [1]. Adaptive radiations and explosive diversification of species [2] are frequently characterized by interspecific phenotypic differences in divergence of few, hypervariable phenotypic traits [3–6]. Such systems offer exceptional advantages to study the genomic bases of phenotypic diversity: they can provide replication under a controlled phylogenetic framework [7], and couple ample phenotypic differentiation with relatively “clean” genomic signals between recently diverged species [8]. Study of the genomic mechanisms underlying hypervariable phenotypic traits has identified, in some cases, relatively simple genetic architectures [9–13]. More often, the genetic architecture underlying such traits can be complex and polygenic [14–17]. It has long been recognized that changes in gene expression can affect phenotypic differences between species [18], and RNA-seq based approaches have greatly facilitated the study of this relationship [19]. A growing number of studies have examined differences in gene expression in phenotypic evolution (e.g., [19–27]). While these studies do not investigate mutational causes, analysis of differential gene expression (DGE) can be a useful approach in examining the genomic basis of divergent phenotypes.

African weakly electric fish (Teleostei: Mormyridae) are among the most rapidly speciating groups of ray-finned fishes [28, 29]. This is partly due to the diversification of the genus *Paramormyrops* [30, 31] in the watersheds of West-Central Africa, where more than 20 estimated species [32] have evolved within the last 0.5–2 million years [30]. Extensive evidence has demonstrated that electric organ discharges (EODs) exhibit little intraspecific variation, yet differ substantially among mormyrid species [33–35]. This pattern is particularly evident in *Paramormyrops* [30, 36], in which EOD waveforms evolve much faster than morphology, size, and trophic ecology [37].

**Fig. 1:**
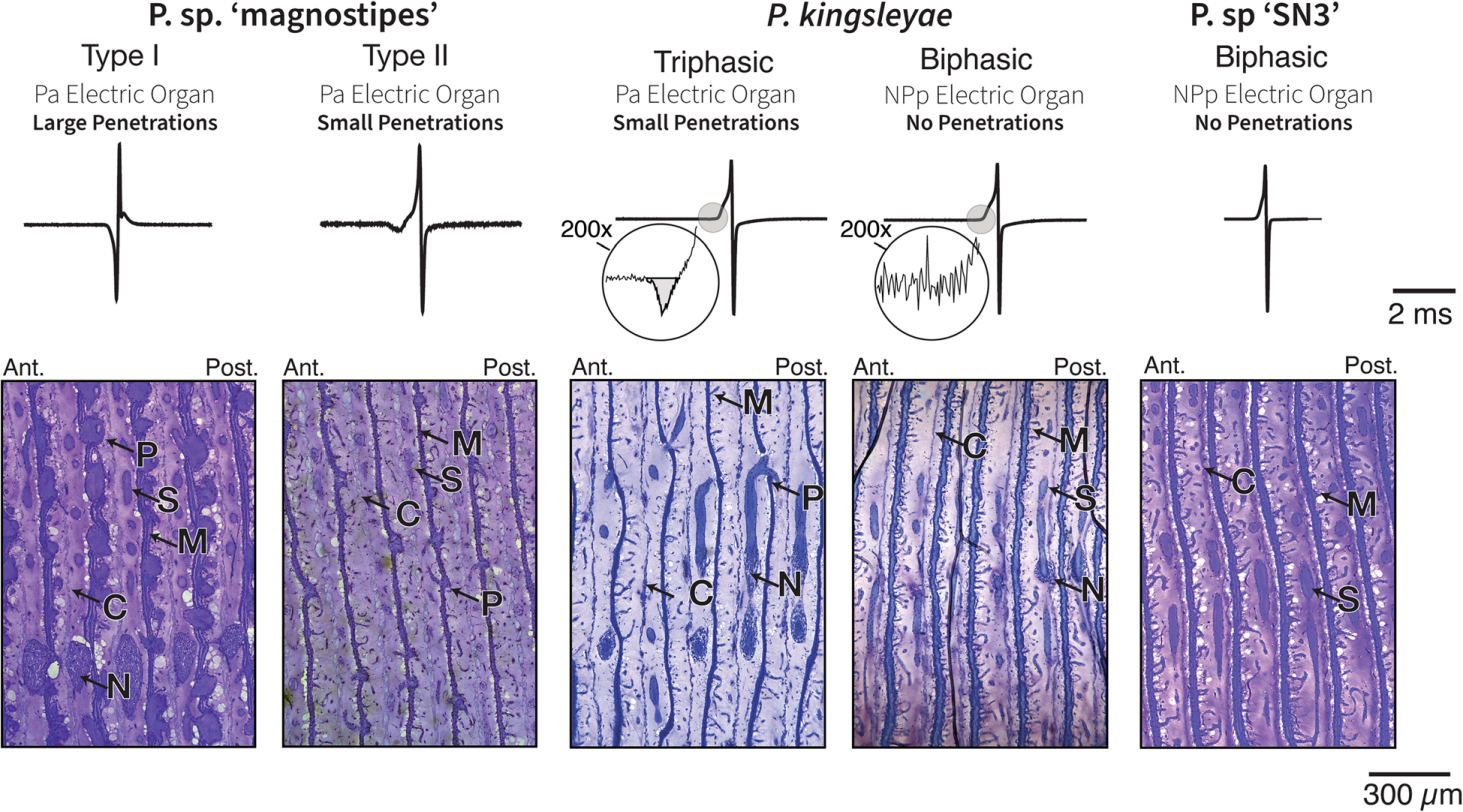
Electric organ discharge (EOD) diversity and electric organ anatomy in *Paramormyrops*. EOD traces from specimens in this study and representative parasagittal sections of the five *Paramormyrops* operational taxonomic units (OTUs) considered in this study. 200x magnification on *P. kingsleyae* EODs reveals a P0 phase on triphasic EODs only. Individuals with triphasic EODs all have penetrations, whereas individuals with biphasic EODs do not. OTUs with ‘inverted’ polarity triphasic EODs have large penetrations compared to OTUs with normal polarity triphasic EODs. Ant. = anterior, C = connective tissue septa, N = nerve, NPp = non-penetrating, posteriorly innervated stalks, M = microstalklets (profusely branched stalks), P = penetrations, Pa = penetrating, anteriorly innervated stalks, Post. = posterior, S = stalks. Image from *P. kingsleyae* biphasic originally appeared in reference 43

Mormyrid EODs are a behavior with a dual role in electrolocation [38, 39] and intraspecific communication [40, 41]. EOD waveforms vary between species principally in terms of their complexity, polarity, and duration [30, 42], and all three dimensions of variation are evident among *Paramormyrops* (Fig. 1). Furthermore, recent discoveries of intraspecific polymorphism in EOD waveform in *P. kingsleyae* [43] and polarity among *P.* sp. ‘magnostipes’ [35] present a unique opportunity to study the genomic basis of phenotypic traits within a rapidly diverging species group.

EODs have a well-understood morphological (Fig. 1) and neurophysiological basis [44, 45]. EODs are generated by specialized cells (electrocytes) that constitute the electric organ (EO), located in the caudal peduncle [46]. Mormyrid EOs are comprised of 80–360 electrocytes [34], and an individual EOD is produced when the electrocytes discharge synchronously. EODs are multiphasic because they result from action potentials produced by two excitable membranes: the two large phases of the EOD, called P1 and P2, are produced by spikes generated by the posterior and anterior electrocyte faces, respectively [47]. There is a relationship between EODs of longer duration and increased surface membrane area [48], likely mediated at least in part by an increase in membrane capacitance [49, 50]. The duration of EODs is highly variable within mormyrids-- some EODs are extremely long (> 15 ms) and others are very brief (0.2 ms) [32].

Within the Mormyridae, triphasic EODs evolved early from biphasic EODs; however, there have been multiple parallel reversions to biphasic EODs across mormyrids and within *Paramormyrops* [36, 43]. Triphasic (P0-present) EODs are produced by electrocytes that are innervated on the anterior face and have penetrating stalks (*Pa*, P-type), whereas biphasic (P0-absent) EODs are produced by electrocytes innervated on the posterior face and lack penetrating stalks (*NPp*, N-type) (for more details see [42, 43, 47, 48, 51, 52]). We refer to triphasic EODs as more ‘complex’ than biphasic EODs. In some cases, triphasic EODs display an unusually large P0 phase, which gives the appearance of an ‘inverted’ polarity. This is exemplified by the type I EODs of *P.* sp. ‘magnostipes’ (Fig. 1) [35]. The number [47] and diameter [34, 43] of stalk penetrations are positively correlated with the magnitude of P0. We refer to individuals with large penetrations as ‘inverted’ polarity and individuals with small penetrations as ‘normal’ polarity.

Recent studies in mormyrids [53–57] have adopted a candidate gene approach to examine the molecular basis of variation in EOD duration on macroevolutionary scales, implicating voltage gated sodium channels (e.g. *scn4aa*) and potassium channels (e.g. *kcna7a*) as key targets of selection during EOD evolution. Beyond this recent attention to ion channels, several studies have described the importance of structural differences between EOs as an important component of EOD variation [43, 48, 50]. In this study, we took a transcriptome-wide approach to characterizing the molecular basis of electric signal diversity in *Paramormyrops* species divergent for EOD complexity, duration and polarity. We used RNA-sequencing to comprehensively examine DGE in the adult EOs of five *Paramormyrops* operational taxonomic units (OTUs)*,* leveraging a recently sequenced and annotated genome assembly from the species *P. kingsleyae* (N-type) [58], and identify gene expression correlates of each of the three main EOD waveform features of electric signal diversity in *Paramormyrops*. Our results emphasize genes that influence the shape and structure of the electrocyte cytoskeleton, membrane and extracellular matrix (ECM) to exhibit predictable differences between *Paramormyrops* species with divergent EOD phenotypes.

## Results

### Overall results

We extracted and sequenced mRNA from EOs of 11 wild caught *Paramormyrops* samples from five OTUs (Table 1). Overall alignment rates of the processed reads to the *Paramormyrops kingsleyae* reference transcriptome ranged from 28 to 74% (> 375 million sequenced reads in total, 50% aligned), with no clear differences among OTUs (Additional file 1). On inspection, we concluded that these rates are a consequence of the presence of overrepresented sequences from rRNA, mtDNA and bacterial contamination in the RNA-seq reads.

**Table 1:** Phenotypic and collection information of the samples studied

Tag No.	OTU	Phenotypes per EOD feature			CUMV Accession Number	Site Name	Lat, Long	SL (mm)	Sex
		Duration	Complexity	Polarity (diameter of penetrations)					
PKINGN_6898	*P. kingsleyae* (N-type)	long EOD	biphasic	NA (no penetrations)	95184	Bikagala Creek	−2.20, 11.56	131	M
PKINGN_6900	*P. kingsleyae* (N-type)	long EOD	biphasic	NA (no penetrations)	95184	Bikagala Creek	−2.20, 11.56	145	M
PKINGN_6901	*P. kingsleyae* (N-type)	long EOD	biphasic	NA (no penetrations)	95184	Bikagala Creek	−2.20, 11.56	149	M
PKINGP_6716	*P. kingsleyae* (P-type)	long EOD	triphasic	small penetrations	95183	Mouvanga Creek	−2.33, 11.69	92.5	J/F
PKINGP_6718	*P. kingsleyae* (P-type)	long EOD	triphasic	small penetrations	95183	Mouvanga Creek	−2.33, 11.69	91	J/F
PMAG1_6780	*P.* sp. ‘magnostipes type I’	long EOD	triphasic	large penetrations	95155	Mouvanga Creek	−2.33, 11.69	107	M
PMAG1_6787	*P.* sp. ‘magnostipes type I’	long EOD	triphasic	large penetrations	95155	Mouvanga Creek	−2.33, 11.69	97.5	F
PMAG2_6768	*P.* sp. ‘magnostipes type II’	long EOD	triphasic	small penetrations	95155	Mouvanga Creek	−2.33, 11.69	93	M
PMAG2_6769	*P.* sp. ‘magnostipes type II’	long EOD	triphasic	small penetrations	95155	Mouvanga Creek	−2.33, 11.69	124	M
PSN3_6739	*P.* sp. ‘SN3’	short EOD	biphasic	NA (no penetrations)	Uncatalogued	Mouvanga Creek	−2.33, 11.69	73	J/F
PSN3_6742	*P.* sp. ‘SN3’	short EOD	biphasic	NA (no penetrations)	95173	Mouvanga Creek	−2.33, 11.69	70	J/F

*CUMV* Cornell University Museum of Vertebrates, *EOD* electric organ discharge, *F* female, *J* juvenile, *Lat* Latitude, *Long* Longitude, *M* male, *NA* not applicable, *OTU* operational taxonomic unit, *SL* standard length

**Fig. 2:**
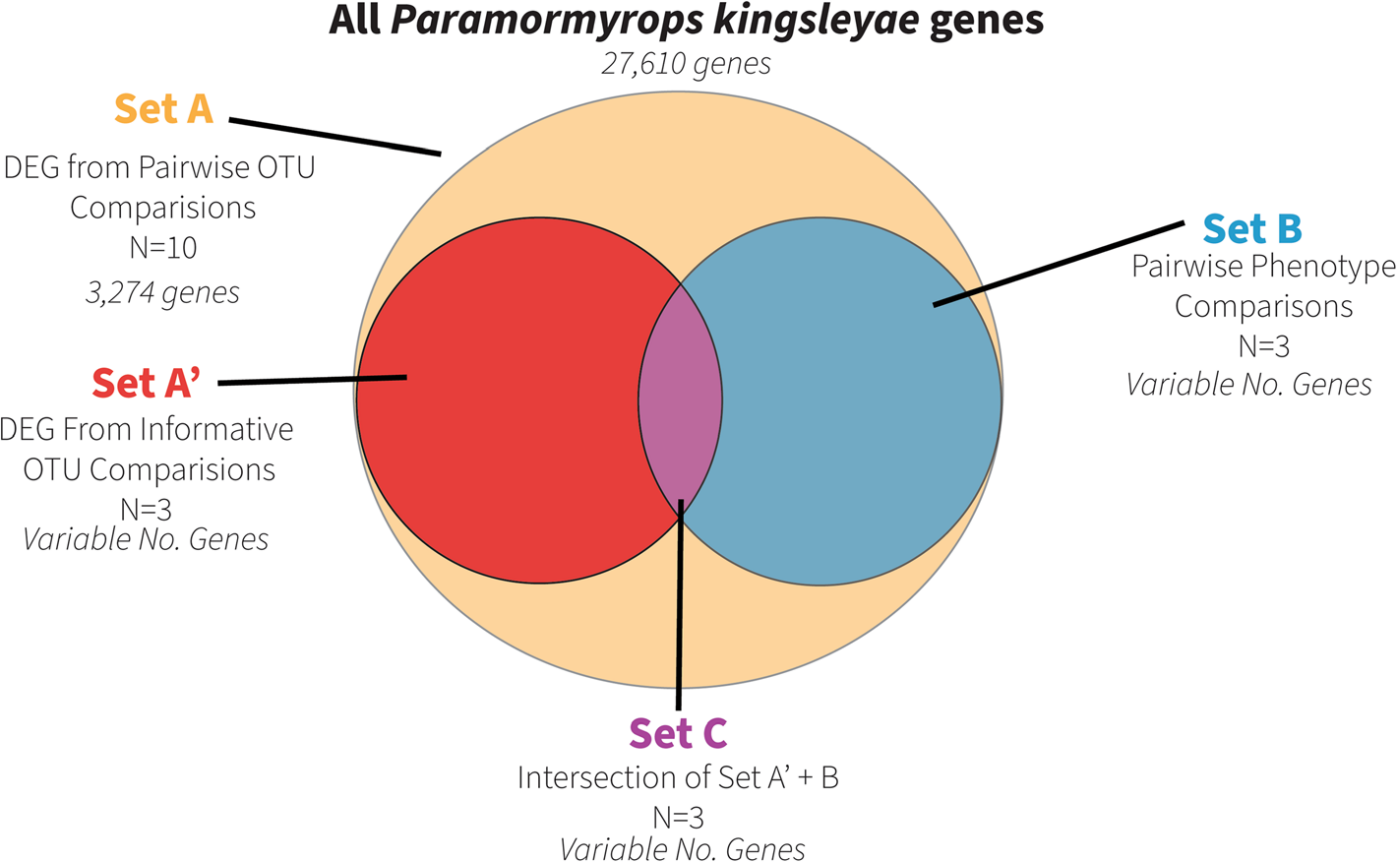
Heatmap of sample by sample correlations in gene expression, and the inferred relationships among OTUs from these expression correlation values. OTU = operational taxonomic unit

We explored the data with a heatmap of pairwise correlations of gene expression for 24,960 genes across all 11 samples (Fig. 2), and carried out all possible pairwise DGE comparisons of OTUs (*n* = 10, Table 2). These ten comparisons detected a range of 16,420–19,273 expressed genes. Intersection of these lists resulted in a non-redundant list of 20,197 genes expressed in EO across all DGE comparisons. We found that 3274 (16%) were differentially expressed in at least one comparison, and expression patterns across all OTUs were highly correlated (Pearson’s r > 0.89, Fig. 2). Despite this, correlation values were higher within recognized OTUs, except for the *P.* sp. ‘magnostipes type II’ 6768 sample (Fig. 2). Thus, we did not use *P.* sp. ‘magnostipes type I’ vs *P.* sp. ‘magnostipes type II’ as the informative comparison for waveform polarity in Set A’ (see methods).

**Table 2:** All ten possible pairwise DGE comparisons with the total number of DEG and enriched GO terms for each. Also indicated is whether each comparison is informative for contrasting each EOD feature. The phenotypes for waveform polarity can only be contrasted in comparisons where both OTUs have penetrations. Informative comparisons for each EOD feature (Set A’) are marked with an * in the column of the EOD feature they contrasted

Comparison		Contrast			DE Genes	enriched GO terms		
OTU #1	OTU #2	Duration	Complexity	Polarity		BP	CC	MF
*P. kingsleyae* (N-type)	*P. kingsleyae* (P-type)	no	yes*	NA (no)	530	46	12	17
*P. kingsleyae* (N-type)	*P.* sp. ‘magnostipes type I’	no	yes	NA (no)	1542	76	15	37
*P. kingsleyae* (N-type)	*P.* sp. ‘magnostipes type II’	no	yes	NA (no)	1174	71	16	25
*P. kingsleyae* (N-type)	*P.* sp. ‘SN3’	yes*	no	NA (no)	507	52	4	13
*P. kingsleyae* (P-type)	*P.* sp. ‘magnostipes type I’	no	no	yes*	1322	40	12	25
*P. kingsleyae* (P-type)	*P.* sp. ‘magnostipes type II’	no	no	no	719	47	10	24
*P. kingsleyae* (P-type)	*P.* sp. ‘SN3’	yes	yes	NA (no)	385	33	3	14
*P.* sp. ‘magnostipes type I’	*P.* sp. ‘magnostipes type II’	no	no	yes	9	5	1	1
*P.* sp. ‘magnostipes type I’	*P.* sp. ‘SN3’	yes	yes	NA (no)	1053	43	6	27
*P.* sp. ‘magnostipes type II’	*P.* sp. ‘SN3’	yes	yes	NA (no)	489	40	9	16

*BP* biological process, *CC* cellular component, *DEG* differentially expressed genes, *DGE* differential gene expression, *GO* gene ontology, *NA* not applicable, *MF* molecular function, *OTU* operational taxonomic unit

### Set A: differential expression analysis

**Fig. 3:**
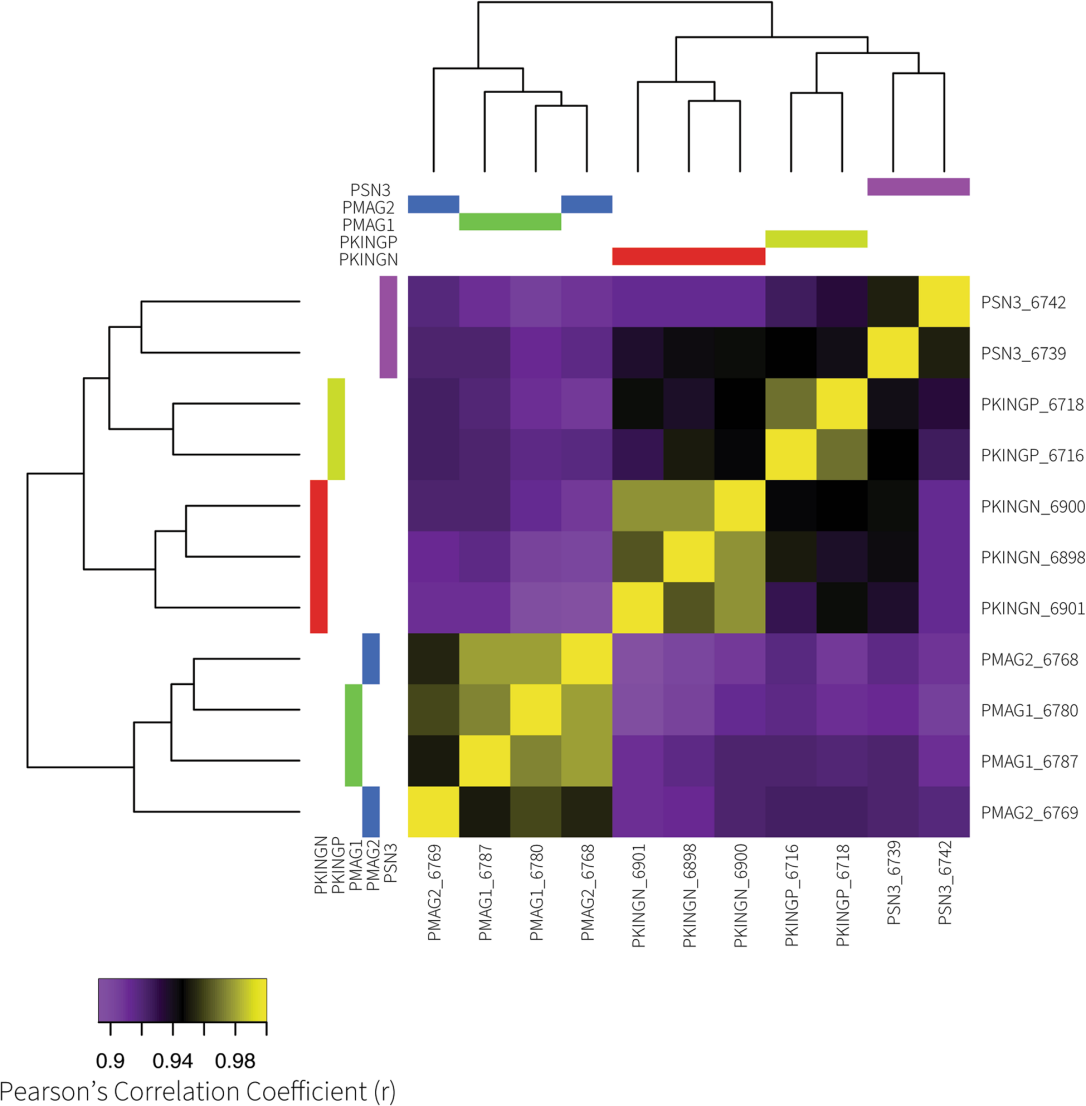
Diagram of how we constructed the lists of upregulated genes of Set C. DEG = differentially expressed genes, N = number of comparisons made for each set, OTU = operational taxonomic unit

We found between 489 and 1542 differentially expressed genes (DEGs; fold change > 4, FDR-corrected *p*-value < 0.001) (50–128 enriched Gene Ontology (GO) terms) in every pairwise comparison of OTUs except *P.* sp. ‘magnostipes type I’ vs *P.* sp. ‘magnostipes type II’, which had only nine DEGs with seven enriched GO terms (Table 2). Additional file 2 provides a tabular list of DEGs for each comparison, and Additional file 3 provides a tabular list of enriched GO terms for each comparison. We call Set A the non-redundant list of 3274 genes that were differentially expressed in at least one DGE comparison (Fig. 3, Set A).

We chose the phylogenetically most informative comparisons to construct Set A’, which are indicated in Table 2. We found: 507 DEG and 69 enriched GO terms comparing *P. kingsleyae* (N-type) vs *P.* sp. ‘SN3’ (EOD duration); 1322 DEG and 77 enriched GO terms comparing *P. kingsleyae* (P-type) vs *P.* sp. ‘magnostipes type I’ (waveform polarity); and 530 DEG and 75 enriched GO terms comparing *P. kingsleyae* (N-type) vs *P. kingsleyae* (P-type) (waveform complexity).

### Set B: expression based clustering

For each EOD feature (*n* = 3), we grouped OTUs by phenotype (Table 1), and calculated normalized expression values for Set A genes. From these, we constructed Set B by selecting genes that (1) exhibit greater than four-fold difference in the average expression levels between phenotypes of each EOD feature, and (2) have within-phenotype standard deviations less than the difference between phenotype-mean expression. For the phenotypes of waveform duration, we identified a combined total of 309 DEG and 43 enriched GO terms, for waveform polarity we found 169 DEG and 14 enriched GO terms, and for waveform complexity the totals were 413 DEG and 38 enriched GO terms. Additional file 4 lists the identities of these DEG and Additional file 5 lists their enriched GO terms for all three GO ontologies.

### Set C: intersection of phylogenetically informative comparisons and expression based clustering

**Fig. 4:**
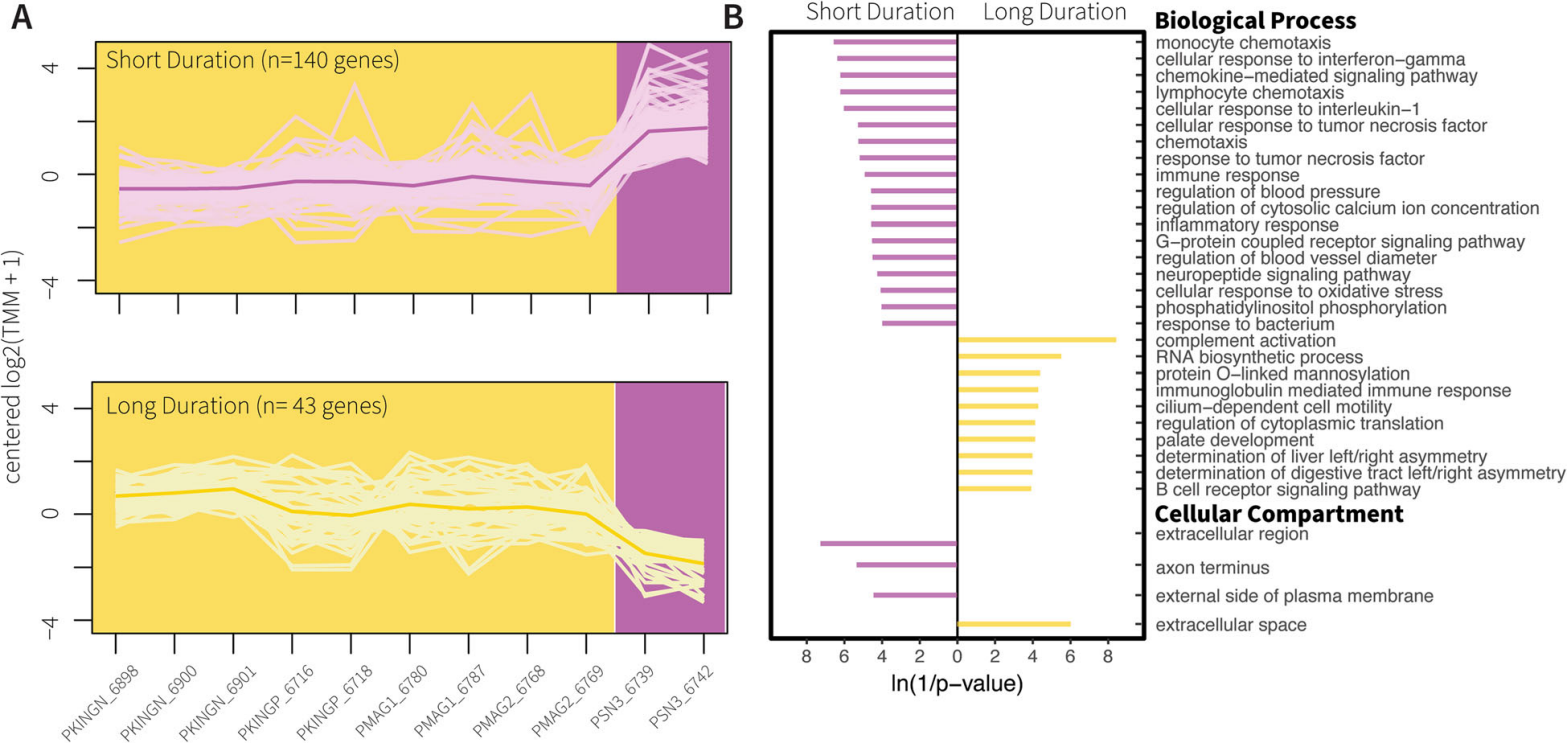
Set C for waveform duration. **a**) Expression patterns of Set C genes for the waveform duration phenotypes short EODs (purple background) and long EODs (yellow background). Samples are sorted alphabetically on the X axis. The lines connect transformed gene expression values across all samples; light-color lines represent one gene, the dark-color line is the average expression pattern of all genes. **b**) Gene Ontology (GO) terms for Biological Process and Cellular Component found enriched in the gene lists from (**a**). The X axis shows transformed *p*-values, the longer a bar the smaller its p-value. The direction and color of a bar indicate the phenotype in which the GO term is enriched [same color code as (**a**)]

**Fig. 5:**
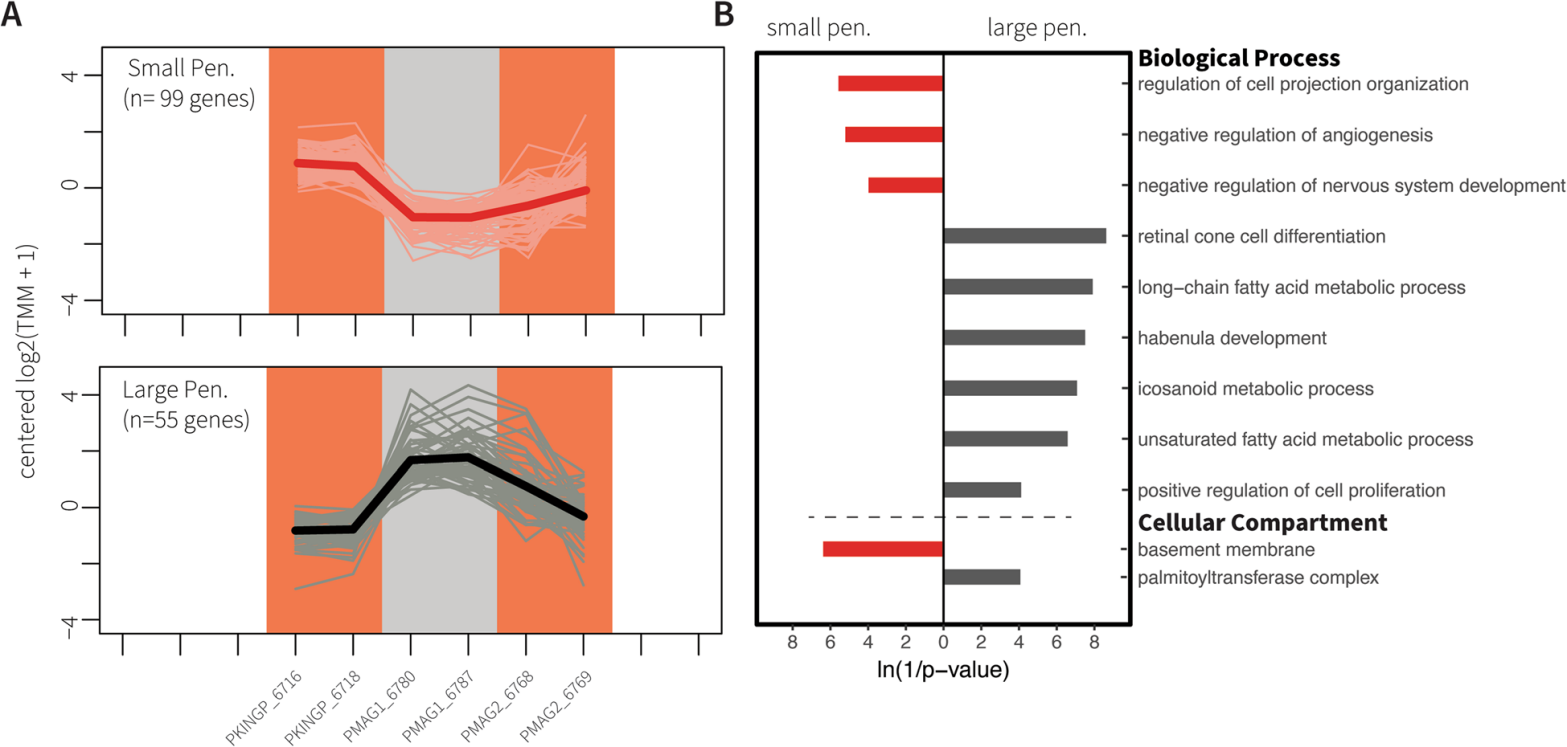
Set C for waveform polarity. **a**) Expression patterns of Set C genes for the waveform polarity phenotypes small penetrations (red background) and large penetrations (grey background). Samples are sorted alphabetically on the X axis. The lines connect transformed gene expression values across all samples; light-color lines represent one gene, the dark-color line is the average expression pattern of all genes. **b**) Gene Ontology (GO) terms for Biological Process and Cellular Component found enriched in the gene lists from (**a**). The X axis shows transformed *p*-values, the longer a bar the smaller its p-value. The direction and color of a bar indicate the phenotype in which the GO term is enriched [same color code as (**a**)]. Pen = penetrations

**Fig. 6:**
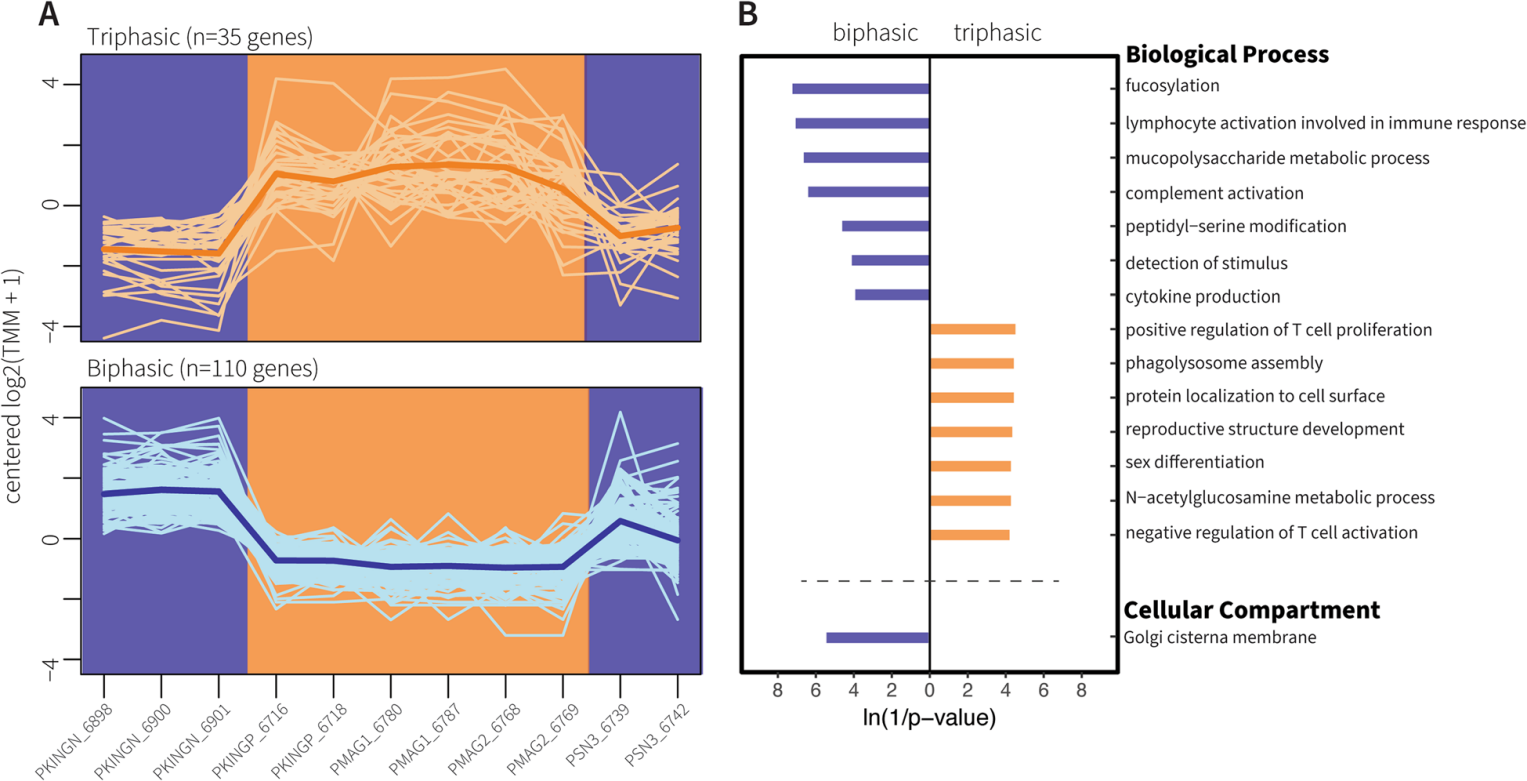
Set C for waveform complexity. **a**) Expression patterns of Set C genes for the waveform complexity phenotypes triphasic (orange background) and biphasic (blue background). Samples are sorted alphabetically on the X axis. The lines connect transformed gene expression values across all samples; light-color lines represent one gene, the dark-color line is the average expression pattern of all genes. **b**) Gene Ontology (GO) terms for Biological Process and Cellular Component found enriched in the gene lists from (**a**). The X axis shows transformed p-values, the longer a bar the smaller its p-value. The direction and color of a bar indicate the phenotype in which the GO term is enriched [same color code as (**a**)]

We were motivated to obtain the DEGs and enriched GO terms that were most likely to be associated with divergent EOD phenotypes. To obtain this list, we constructed Set C, which is the intersection of Set A’ and Set B (Fig. 3, see methods). The expression profiles of the Set C genes for each EOD feature, along with the enriched GO terms for Biological Process and Cellular Component, are shown in Figs. 4-6.

Contrast of waveform duration identified 183 DEG and 39 enriched GO terms. 140 of the DEG were upregulated in the short EOD phenotype (Fig. 4 purple lines), and 43 genes were upregulated in samples with long EODs (Fig. 4 yellow lines). Contrast of waveform polarity identified 154 DEG and 17 enriched GO terms. We found 99 upregulated genes in individuals with small penetrations (Fig. 5 red lines), and 55 upregulated genes in individuals with large penetrations (Fig. 5 grey lines). Finally, contrast of waveform complexity identified 145 DEG and 20 enriched GO terms. We detected 110 upregulated genes in individuals with biphasic EODs (Fig. 6 blue lines), and 35 upregulated genes in individuals with triphasic EODs (Fig. 6 orange lines). These results are further detailed in Table 3. All genes in Set C are listed in Additional file 6 and their enriched GO terms are listed in Additional file 7.

**Table 3:** Total number of upregulated genes and enriched GO terms in Set C for each EOD feature, phenotype and ontology

EOD feature	Phenotype	Upregulated genes	Protein-coding (%)	enriched GO terms		
				BP	CC	MF
Duration	short EODs	140	122 (87)	18	3	5
Duration	long EODs	43	34 (79)	10	1	2
Polarity	small penetrations	99	89 (90)	3	1	3
Polarity	large penetrations	55	40 (73)	6	1	3
Complexity	biphasic	110	81 (74)	7	1	2
Complexity	triphasic	35	25 (71)	7	0	3

*BP* biological process, *CC* cellular component, *EOD* electric organ discharge, *GO* gene ontology, *MF* molecular function

## Discussion

It has long been recognized that changes in gene expression can affect phenotypic differences between species [18], and RNA-seq has facilitated the study of this relationship [19]. The goal of this study was to determine DEGs associated with divergent EOD features within *Paramormyrops*. Expression patterns across all OTUs were highly correlated (Pearson’s r > 0.89, Fig. 2) and we detected differential expression of only 3274 (16%) genes between any two OTUs. Thus, a major finding of this study is that EO gene expression is overall quite similar across *Paramormyrops* species with divergent EODs, and relatively few genes are associated with phenotypic differences in EOD waveform between OTUs. We find this notable given observations of generally high levels of genetic distances between geographically proximate populations of the same *Paramormyrops* species [35, 59].

Despite the relatively small number of DEGs compared to the total number of genes expressed in the EO, we constructed our analysis to extract genes that were highly associated with particular phenotypes. Set A’ represents a formal statistical test that contrasted OTUs. Each comparison contrasted samples from OTUs that were divergent in only one EOD feature, while minimizing phylogenetic distance. The drawback of this approach is the observed differences may not reflect a general pattern across multiple OTUs, instead resulting from OTU-specific changes or confounding variables such as collection sites. Set B took the opposite approach, using information from all possible biological replicates to identify consistent gene expression patterns between EOD phenotypes, at the expense of formal statistical support and the introduction of confounding phylogenetic relationships and phenotypic heterogeneity. To balance these drawbacks, we constructed Set C, which represents genes and GO terms that are differentially expressed/enriched between closely related OTUs divergent in only one phenotypic character and that are also consistently differentially expressed/enriched among representatives with similar EOD phenotypes. As such, we focus our discussion on the results of Set C. We classified the genes in Set C into “general” functional classes, or themes; and focus our attention on the ones that relate to the known morphological underpinnings of waveform duration (Table 4), polarity (Table 5), and complexity (Table 6). These functional classes were genes related to the ECM, cation homeostasis, lipid metabolism, and cytoskeletal and sarcomeric genes.

**Table 4:** Selected DEG in Set C for waveform duration by “general” functional class and EOD phenotype, and highlights of their predicted function

NCBI Gene ID	Gene Description	Gene Symbol	“general” functional class	upregulated in phenotype	Highlights of Predicted Function (edited from UniProt)
111834716	*Kv channel-interacting protein 4*	LOC111834716	Cation homeostasis	short EODs	Regulatory subunit of Kv4/D (Shal)-type voltage-gated rapidly inactivating A-type potassium channels. Regulates channel density, inactivation kinetics and rate of recovery from inactivation in a calcium-dependent and isoform-specific manner
111836747	*potassium channel subfamily K member 5-like*	LOC111836747	Cation homeostasis	short EODs	pH-dependent, voltage insensitive, outwardly rectifying potassium channel. Outward rectification is lost at high external K+ concentrations
111857989	*chloride intracellular channel protein 5-like*	LOC111857989	Cation homeostasis	short EODs	Can insert into membranes and form poorly selective ion channels that may also transport chloride ions. May play a role in the regulation of transepithelial ion absorption and secretion
111833088	*myosin-7-like*	LOC111833088	Cytoskeletal & sarcomeric	long EODs	Myosins are actin-based motor molecules with ATPase activity essential for muscle contraction
111856289	*pleckstrin homology-like domain family B member 1*	LOC111856289	Cytoskeletal & sarcomeric	long EODs	GO BP: regulation of microtubule cytoskeleton organization
111842483	*parvalbumin-2*	LOC111842483	Cytoskeletal & sarcomeric	short EODs	In muscle, parvalbumin is thought to be involved in relaxation after contraction. It binds two calcium ions
111846153	*troponin I, slow skeletal muscle-like*	LOC111846153	Cytoskeletal & sarcomeric	short EODs	Inhibitory subunit of troponin, the thin filament regulatory complex which confers calcium-sensitivity to striated muscle actomyosin ATPase activity
111851695	*keratin, type II cytoskeletal 8-like*	LOC111851695	Cytoskeletal & sarcomeric	short EODs	Together with KRT19, helps to link the contractile apparatus to dystrophin at the costameres of striated muscle
111856036	*parvalbumin-2-like*	LOC111856036	Cytoskeletal & sarcomeric	short EODs	In muscle, parvalbumin is thought to be involved in relaxation after contraction. It binds two calcium ions
111860236	*tropomyosin alpha-1 chain-like*	LOC111860236	Cytoskeletal & sarcomeric	short EODs	Binds to actin filaments in muscle and non-muscle cells. Plays a central role, in association with the troponin complex, in the calcium dependent regulation of vertebrate striated muscle contraction. In non-muscle cells is implicated in stabilizing cytoskeleton actin filaments.
111845490	*matrilin 2*	*matn2*	Extracellular matrix	long EODs	Involved in matrix assembly
111860169	*thrombospondin-4-like*	LOC111860169	Extracellular matrix	long EODs	Adhesive glycoprotein that mediates cell-to-cell and cell-to-matrix interactions and is involved in various processes including cellular proliferation, migration, adhesion and attachment
111860877	*collagenase 3-like*	LOC111860877	Extracellular matrix	short EODs	Plays a role in the degradation of extracellular matrix proteins
111834720	*protein EFR3 homolog B-like*	LOC111834720	Lipid metabolism	short EODs	Component of a complex required to localize phosphatidylinositol 4-kinase (PI4K) to the plasma membrane. The complex acts as a regulator of phosphatidylinositol 4-phosphate (PtdIns4P) synthesis
111840357	*PTB domain-containing engulfment adapter protein 1-like*	LOC111840357	Lipid metabolism	short EODs	Modulates cellular glycosphingolipid and cholesterol transport
111846286	*retinoic acid receptor responder protein 3-like*	LOC111846286	Lipid metabolism	short EODs	Catalyzes the calcium-independent hydrolysis of acyl groups in various phosphatidylcholines (PC) and phosphatidylethanolamine (PE)
111847640	*phosphatidylinositol 3-kinase regulatory subunit gamma-like*	LOC111847640	Lipid metabolism	short EODs	Binds to activated (phosphorylated) protein-tyrosine kinases through its SH2 domain and regulates their kinase activity
111852373	*proto-oncogene c-Fos-like*	LOC111852373	Lipid metabolism	short EODs	In growing cells, activates phospholipid synthesis, possibly by activating CDS1 and PI4K2A
111857713	*HRAS-like suppressor 3*	LOC111857713	Lipid metabolism	short EODs	Catalyzes the calcium-independent hydrolysis of acyl groups in various phosphatidylcholines (PC) and phosphatidylethanolamine (PE)
111860935	*fatty aldehyde dehydrogenase-like*	LOC111860935	Lipid metabolism	short EODs	Catalyzes the oxidation of medium and long chain aliphatic aldehydes to fatty acids

*BP* biological process, *DEG* differentially expressed genes, *EOD* electric organ discharge, *GO* gene ontology, *NCBI* National Center for Biotechnology Information

**Table 5:** Selected DEG in Set C for waveform polarity, by “general” functional class and EOD phenotype, and highlights of their expected function

NCBI Gene ID	Gene Description	Gene Symbol	“general” functional class	upregulated in phenotype	Highlights of Predicted Function (edited from UniProt)
111840706	*G protein-activated inward rectifier potassium channel 1*	LOC111840706	Cation homeostasis	Small penetrations	This potassium channel is controlled by G proteins. Plays a crucial role in regulating the heartbeat
111853690	*protein kinase cGMP-dependent 1*	*prkg1*	Cation homeostasis	Small penetrations	Serine/threonine protein kinase. Numerous protein targets for PRKG1 phosphorylation are implicated in modulating cellular calcium. Proteins that are phosphorylated by PRKG1 regulate platelet activation and adhesion, smooth muscle contraction, cardiac function, gene expression
111843447	*myosin light chain 4-like*	LOC111843447	Cytoskeletal & sarcomeric	Large penetrations	Regulatory light chain of myosin. Does not bind calcium
111851664	*leucine rich repeat containing 10*	*lrrc10*	Cytoskeletal & sarcomeric	Large penetrations	May play important roles in cardiac development and/or cardiac function
111856907	*Wiskott-Aldrich syndrome protein family member 3-like*	LOC111856907	Cytoskeletal & sarcomeric	Large penetrations	Plays a role in the regulation of cell morphology and cytoskeletal organization. Required in the control of cell shape
111834243	*desmin-like*	LOC111834243	Cytoskeletal & sarcomeric	Small penetrations	Muscle-specific type III intermediate filament essential for proper muscular structure and function. Plays a crucial role in maintaining the structure of sarcomeres. May act as a sarcomeric microtubule-anchoring protein
111843225	*protein kinase C alpha type-like*	LOC111843225	Cytoskeletal & sarcomeric	Small penetrations	Regulates cardiomyocyte function by phosphorylating cardiac troponin T (TNNT2/CTNT), which induces significant reduction in actomyosin ATPase activity, myofilament calcium sensitivity and myocardial contractility
111848393	*PH and SEC7 domain-containing protein 1-like*	LOC111848393	Cytoskeletal & sarcomeric	Small penetrations	Induces cytoskeletal remodeling
111849608	*CDC42 effector protein 3*	*cdc42ep3*	Cytoskeletal & sarcomeric	Small penetrations	Probably involved in the organization of the actin cytoskeleton. May act downstream of CDC42 to induce actin filament assembly leading to cell shape changes
111853190	*protein phosphatase 1 regulatory subunit 12B-like*	LOC111853190	Cytoskeletal & sarcomeric	Small penetrations	Regulates myosin phosphatase activity. Augments Ca2+ sensitivity of the contractile apparatus
111856340	*calponin-1-like*	LOC111856340	Cytoskeletal & sarcomeric	Small penetrations	Thin filament-associated protein that is implicated in the regulation and modulation of smooth muscle contraction. It is capable of binding to actin, calmodulin, troponin C and tropomyosin. The interaction of calponin with actin inhibits the actomyosin Mg-ATPase activity
111856797	*myosin light chain 3-like*	LOC111856797	Cytoskeletal & sarcomeric	Small penetrations	Regulatory light chain of myosin. Does not bind calcium
111838718	*inter-alpha-trypsin inhibitor heavy chain H5-like*	LOC111838718	Extracellular matrix	Small penetrations	inter-alpha-trypsin inhibitors usually interact with hyaluronan
111841241	*epiphycan-like*	LOC111841241	Extracellular matrix	Small penetrations	May have a role in bone formation and also in establishing the ordered structure of cartilage through matrix organization
111844627	*collagen alpha-1(V) chain-like*	LOC111844627	Extracellular matrix	Small penetrations	Type V collagen is a member of group I collagen (fibrillar forming collagen). It is a minor connective tissue component of nearly ubiquitous distribution. Type V collagen binds to DNA, heparan sulfate, thrombospondin, heparin, and insulin
111853425	*thrombospondin 2*	*thbs2*	Extracellular matrix	Small penetrations	Adhesive glycoprotein that mediates cell-to-cell and cell-to-matrix interactions
111854264	*collagen type IX alpha 2 chain*	*col9a2*	Extracellular matrix	Small penetrations	Structural component of hyaline cartilage and vitreous of the eye
111857302	*anthrax toxin receptor 1-like*	LOC111857302	Extracellular matrix	Small penetrations	Interacts with extracellular matrix proteins and with the actin cytoskeleton. Mediates adhesion of cells to type 1 collagen and gelatin, reorganization of the actin cytoskeleton and promotes cell spreading
111857834	*collagen alpha-4(VI) chain-like*	LOC111857834	Extracellular matrix	Small penetrations	Collagen VI acts as a cell-binding protein
111859912	*cell migration-inducing and hyaluronan-binding protein-like*	LOC111859912	Extracellular matrix	Small penetrations	Mediates depolymerization of hyaluronic acid (HA) via the cell membrane-associated clathrin-coated pit endocytic pathway. Binds to hyaluronic acid
111834720	*protein EFR3 homolog B-like*	LOC111834720	Lipid metabolism	Large penetrations	Component of a complex required to localize phosphatidylinositol 4-kinase (PI4K) to the plasma membrane. The complex acts as a regulator of phosphatidylinositol 4-phosphate (PtdIns4P) synthesis
111840084	*cytosolic phospholipase A2-like*	LOC111840084	Lipid metabolism	Small penetrations	Selectively hydrolyzes arachidonyl phospholipids in the sn-2 position releasing arachidonic acid
111853114	*alkaline ceramidase 2-like*	LOC111853114	Lipid metabolism	Small penetrations	Hydrolyzes the sphingolipid ceramide into sphingosine and free fatty acid

*DEG* differentially expressed genes, *EOD* electric organ discharge, *NCBI* National Center for Biotechnology Information

**Table 6:** Selected DEG in Set C for waveform complexity, by “general” functional class and EOD phenotype, and highlights of their expected function

NCBI Gene ID	Gene Description	Gene Symbol	“general” functional class	upregulated in phenotype	Highlights of Predicted Function (edited from UniProt)
111838181	*solute carrier family 9 member A7*	*slc9a7*	Cation homeostasis	Biphasic	Protein: Sodium/hydrogen exchanger 7. Gene: SLC9A7. Mediates electroneutral exchange of protons for Na + and K+ across endomembranes
111848312	*voltage-dependent calcium channel gamma-1 subunit-like*	LOC111848312	Cation homeostasis	Triphasic	Regulatory subunit of the voltage-gated calcium channel that gives rise to L-type calcium currents in skeletal muscle. Regulates channel inactivation kinetics
111841270	*family with sequence similarity 110 member C*	*fam110c*	Cytoskeletal & sarcomeric	Biphasic	May play a role in microtubule organization
111845832	*5′-AMP-activated protein kinase subunit gamma-2-like*	LOC111845832	Cytoskeletal & sarcomeric	Biphasic	AMP/ATP-binding subunit of AMP-activated protein kinase (AMPK). Acts as a regulator of cellular polarity by remodeling the actin cytoskeleton; probably by indirectly activating myosin
111850616	*transmembrane protein 47-like*	LOC111850616	Cytoskeletal & sarcomeric	Biphasic	Regulates cell junction organization in epithelial cells. May regulate F-actin polymerization
111851223	*FYVE, RhoGEF and PH domain-containing protein 4-like*	LOC111851223	Cytoskeletal & sarcomeric	Biphasic	Plays a role in regulating the actin cytoskeleton and cell shape
111857398	*protein-methionine sulfoxide oxidase mical2b-like*	LOC111857398	Cytoskeletal & sarcomeric	Biphasic	Promotes depolymerization of F-actin
111857697	*leiomodin 2*	*lmod2*	Cytoskeletal & sarcomeric	Biphasic	Mediates nucleation of actin filaments and thereby promotes actin polymerization. Plays a role in the regulation of actin filament length. Required for normal sarcomere organization in the heart, and for normal heart function
111854588	*capping protein regulator and myosin 1 linker 3*	*carmil3*	Cytoskeletal & sarcomeric	Triphasic	No info for CARMIL3, but CARMIL2 is a cell membrane-cytoskeleton-associated protein that plays a role in the regulation of actin polymerization at the barbed end of actin filaments. Enhances actin polymerization
111841398	*inter-alpha-trypsin inhibitor heavy chain 3*	*itih3*	Extracellular matrix	Biphasic	May act as a carrier of hyaluronan in serum or as a binding protein between hyaluronan and other matrix proteins
111841399	*inter-alpha-trypsin inhibitor heavy chain H3-like*	LOC111841399	Extracellular matrix	Biphasic	May act as a carrier of hyaluronan in serum or as a binding protein between hyaluronan and other matrix proteins
111853010	*ependymin-like*	LOC111853010	Extracellular matrix	Triphasic	GO MF: calcium ion binding. GO BP: cell-matrix adhesion
111853814	*epiphycan-like*	LOC111853814	Extracellular matrix	Triphasic	May have a role in bone formation and also in establishing the ordered structure of cartilage through matrix organization

*BP* biological process, *DEG* differentially expressed genes, *EOD* electric organ discharge, *GO* gene ontology, *MF* molecular function, *NCBI* National Center for Biotechnology Information

### Waveform duration

Several researchers have implicated the role of ion channels in the evolution of duration changes in mormyrid signals [53–57]. We did not find evidence of large changes in expression of sodium channels between short-duration *P.* sp. ‘SN3’ and other *Paramormyrops* species; but we detected upregulation in short EOD samples of a voltage insensitive, outwardly rectifying potassium channel (*potassium channel subfamily K member 5-like*) and of a regulatory subunit of a Shal-type voltage-gated potassium channel (*Kv channel-interacting protein 4*). Additionally, individuals with short EODs upregulate two calcium-binding proteins: *parvalbumin-2*, and *parvalbumin-2-like*. Parvalbumins are highly expressed in skeletal muscle where they sequester calcium after contraction, thus facilitating relaxation. Frequently, muscles with fast relaxation rates express higher levels of parvalbumins [60]. The upregulated parvalbumin genes we detected may somehow be related to shorter EODs by sequestering calcium at a faster rate, which could affect action potentials directly or indirectly through calcium-activated ion channels.

Previous studies have demonstrated that changes in EOD duration result from changes in electrocyte ultrastructure. The two major phases of the EOD waveform are caused by action potentials generated by the anterior and posterior faces [47]. Bennett [48] demonstrated a relationship between EOD duration and increased surface membrane area, and Bass et al. [50] showed that differences in surface area are more readily noticeable on the anterior face. Membrane surface area is increased by folding the electrocyte membrane into papillae and other tube-like invaginations [61]. Testosterone can induce increases in EOD duration in several mormyrids [49, 50, 62, 63], and it also increases membrane surface area, either particularly on the anterior face [50] or on both anterior and posterior faces [64]. A larger surface area may increase the capacitance of the membrane, thus delaying spike initiation [49, 50]. Consequently, genes involved in the synthesis of membranes could influence EOD duration.

We found the most prominent differences in gene expression between the EOD duration phenotypes in genes that code for cytoskeletal, sarcomeric, and lipid metabolism proteins (Table 4). We emphasize the last group: no lipid metabolism genes were upregulated in individuals with long EODs, whereas samples with short EODs upregulated *protein EFR3 homolog B-like* (a regulator of phosphatidylinositol 4-phosphate synthesis), *retinoic acid receptor responder protein 3-like* and *HRAS-like suppressor 3* (these two catalyze hydrolysis of phosphatidylcholines and phosphatidylethanolamines), *fatty aldehyde dehydrogenase-like* (fatty acid metabolism), *PTB domain-containing engulfment adapter protein 1-like* (modulates cellular glycosphingolipid and cholesterol transport), *phosphatidylinositol 3-kinase regulatory subunit gamma-like*, (PI3K, which phosphorylates phosphatidylinositol), and *proto-oncogene c-Fos-like* (can activate phospholipid synthesis), and showed enrichment of the GO term ‘phosphatidylinositol phosphorylation.’ We hypothesize that these genes are involved in the surface proliferation of the electrocytes membranes.

Additionally, each mormyrid electrocyte stands embedded in a gelatinous mucopolysaccharide matrix (the ECM) separated from neighboring electrocytes by connective tissue septa (Fig. 1) [34], and the membrane surface invaginations are coated by the same ECM that surrounds the electrocytes [50, 61]. Hence, differences in surface invaginations could also be reflected in differences in the expression of genes whose products interact with the ECM. In individuals with long EODs, we found upregulated the genes *matrilin 2* (involved in matrix assembly) and *thrombospondin-4-like* (mediates cell-to-matrix interactions), and enriched the GO term ‘extracellular space’; whereas those with short EODs upregulated *collagenase 3-like* (plays a role in the degradation of ECM proteins) and displayed enrichment of the GO terms ‘extracellular region’ and ‘external side of plasma membrane’.

Two previous studies focused on DGE between EOs in another mormyrid species adaptive radiation/explosive diversification (genus *Campylomormyrus*, whose EO anatomy closely resembles that of *Paramormyrops*). Both focused on comparisons between species with biphasic EODs but different waveform duration phenotypes: *C. tshokwe* (long duration) and *C. compressirostris* (short duration). The first study performed a canditate gene approach to quantify the expression patterns of 18 sodium and potassium homeostasis genes between the EOs of the two species [55], whereas Lamanna et al. [65] used RNA-seq to simultaneously compare gene expression between EOs of these species. While we did not observe differences in expression of any of the potassium channels reported by Nagel et al. [55], we note that Lamanna et al. [65] reported differential expression of metabolic pathways related genes, particularly fatty acid metabolism, and ion transport and neuronal function (referred to in their text as cross-species analysis (EO) subclusters 2 and 4). While we found no overlap in the identities of any specific genes in our study, we note that our analysis also detected differential expression of lipid metabolism related genes when comparing EODs of different duration.

Overall, our results identify genes that may affect EOD duration through membrane rearrangements, which could be coupled with changes in the interaction with the ECM and the expression of cytoskeletal and sarcomeric genes. Since this waveform feature is modulated by testosterone, this androgen could facilitate the study of these suggested genetic underpinnings under more rigorously controlled circumstances.

### Waveform polarity

The number [47] and diameter [34, 43] of stalk penetrations are positively correlated with the magnitude of P0. This phenomenon is exemplified by *P.* sp. ‘magnostipes type I’, which has the largest P0 in the OTUs examined in this study, giving the EOD the appearance that it ‘inverted’ relative to other EODs. This OTU has numerous, large diameter penetrations, whereas *P. kingsleyae* (P-type) has relatively fewer, small diameter penetrations (Fig. 1). These large structural differences may influence the electrocyte’s connection with the surrounding ECM, and our results support this: the phenotypes of waveform polarity exhibited differences in the expression of genes that interact with the extracellular space. We found no such genes upregulated in individuals with large penetrations, whereas in samples with small penetrations we detected the enriched GO terms: ‘extracellular matrix structural constituent’ and ‘basement membrane,’ and the upregulated genes: *collagen alpha-1(V) chain-like, collagen type IX alpha 2 chain, collagen alpha-4(VI) chain-like, epiphycan-like* (may play a role in cartilage matrix organization), *cell migration-inducing and hyaluronan-binding protein-like* (mediates depolymerization of hyaluronic acid), *inter-alpha-trypsin inhibitor heavy chain H5-like* (although this gene is little studied, inter-alpha-trypsin inhibitors usually interact with hyaluronan), and *thrombospondin 2* (mediates cell-to-matrix interactions).

OTUs with small penetrations also exhibited higher expression of genes related to cytoskeletal, sarcomeric, and lipid metabolism proteins than do individuals with large penetrations (Table 5). This includes the genes *myosin light chain 3-like*, *desmin-like, PH and SEC7 domain-containing protein 1-like* (induces cytoskeletal remodeling), *CDC42 effector protein 3* (probably involved in the organization of the actin cytoskeleton), *protein phosphatase 1 regulatory subunit 12B-like* (regulates myosin phosphatase activity), *protein kinase C alpha type-like* (phosphorylates cardiac troponin T), and *calponin-1-like* (modulates smooth muscle contraction). Samples with large penetrations showed upregulation of the genes *myosin light chain 4-like* (regulatory light chain of myosin), *leucine rich repeat containing 10,* (may play important roles in cardiac development and/or function), and *Wiskott-Aldrich syndrome protein family member 3-like* (regulation of cell morphology and cytoskeletal organization).

We hypothesize that the differences in the number and diameter of penetrations that drive variation in EOD waveform polarity require changes to the electrocyte’s cytoskeletal and membrane properties. These arrangements may be necessary for the electrocytes body to adjust to the increased volume displacements imposed by larger penetrations; or alternatively, they may be a prerequisite for penetrating stalks to enlarge. Our observations support and elaborate on the hypothesis that sarcomeric proteins (which are non-contractile in mormyrid electric organs) may function as a means of cytoskeletal support and structural integrity in mormyrid electrocytes [66].

### Waveform complexity

Waveform complexity refers to the number of phases present in an EOD, and mormyrid EODs vary in the presence of a small head negative phase (P0). The presence or absence of P0 in the EOD depends on the anatomical configuration of the electrocytes: P0-present (or triphasic) EODs are produced by electrocytes that are innervated on the anterior face and have penetrating stalks (Pa), whereas P0-absent (or biphasic) EODs are produced by electrocytes innervated on the posterior face and lack penetrating stalks (NPp) [42, 43, 47, 48, 51, 52]. Developmental studies of the adult EO suggest that Pa electrocytes go through a NPp stage before developing penetrations [67, 68]. This motivated the hypothesis that penetrations develop by the migration of the posteriorly innervated stalk system (NPp stage) through the edge of the electrocyte, and that the interruption of this migration represents a mechanism for Pa-to-NPp reversals [69, 70].

Our data indicates several DEGs that implicate specific cytoskeletal and ECM reorganizations between triphasic and biphasic EODs (Table 6). We observed differential expression of several genes associated with the polymerization of F-actin. In triphasic individuals, we observe upregulation of the gene *capping protein regulator and myosin 1 linker 3* (CARMIL3); although this gene is little studied, its paralog CARMIL2 enhances F-actin polymerization. In contrast, the biphasic phenotype upregulated the genes *protein-methionine sulfoxide oxidase mical2b-like* (promotes F-actin depolymerization), *transmembrane protein 47-like* (may regulate F-actin polymerization), 5′-*AMP-activated protein kinase subunit gamma-2-like* (could remodel the actin cytoskeleton), *FYVE, RhoGEF and PH domain-containing protein 4-like* (regulates the actin cytoskeleton), *leiomodin 2* (promotes actin polymerization, and required for normal sarcomere organization in the heart) and *family with sequence similarity 110 member C* (may play a role in microtubule organization). Thus, biphasic and triphasic EODs display several DEG, with potentially diverging outcomes, that influence the cellular internal structure.

We hypothesize that electrocytes with penetrating stalks (which produce triphasic EODs) require cytoskeletal arrangements to produce penetrations, perhaps related to increasing F-actin, to maintain their structural integrity. Similar to what we propose under waveform polarity, these arrangements may be necessary for the electrocyte body to adjust to the penetrations; or alternatively, they may be a prerequisite for penetrations to occur.

We also observed differential expression in proteins expressed in the ECM. In biphasic OTUs, we found the GO term ‘mucopolysaccharide metabolic process’ to be enriched, and two upregulated copies of the gene *inter-alpha-trypsin inhibitor heavy chain 3*, which may act as a binding protein between hyaluronan and other ECM proteins. In triphasic individuals, we found the upregulated genes *epiphycan-like*, which may play a role in cartilage matrix organization, and *ependymin-like* (ortholog to the zebrafish ependymin-like gene *epdl2*).

Two ependymin-like genes are among the most differentially expressed genes in the Set A’ comparison for waveform complexity *P. kingsleyae* (N-type) vs *P. kingsleyae* (P-type) (500-fold more highly expressed in *P. kingsleyae* (P-type), Additional file 2). Although expressed in many tissues and with little amino acid similarity, all ependymin-related proteins are secretory, calcium-binding glycoproteins that can undergo conformational changes and associate with collagen in the ECM. They have been involved in regeneration, nerve growth, cell contact, adhesion and migration processes [71]. We hypothesize that ependymin-related proteins, and potentially some of the other ECM proteins highly expressed in triphasic individuals, are part of the “fibrillar substance” that lies between the stalk and the electrocyte body in individuals with penetrating electrocytes [50]. Notably, the *P. kingsleyae* genome assembly, which is based on a biphasic individual, contains three paralogs of *epdl2*, whereas the osteoglossiform *Scleropages formosus* only has one, suggesting the intriguing possibility that this gene may have been duplicated in *Paramormyrops* or in mormyrids. Ependymin-related paralogs have been proposed as suitable targets to experimentally test gene subfunctionalization [72].

Altogether, our results for EOD waveform complexity suggest that the conformation of the cytoskeleton and the expression of proteins secreted to the ECM are important elements of the stalk penetrations, which generate triphasic EODs.

## Conclusions

The widespread differential expression within *Paramormyrops* of calcium-related genes (Additional file 6) emphasizes a much-needed area of future research. Calcium is known to be necessary for the proper electrocyte repolarization in some gymnotiform species [73], but it may not be as important in others [74]. Few studies have addressed calcium physiology in mormyrids: calcium-related proteins have been reported as differentially expressed in EO vs skeletal muscle in *Campylomormyrus* [65] and in *Brienomyrus brachyistius* [66]. As electrocytes do not contract, calcium may act in electrocytes as an important second messenger or cofactor, participate in interactions with the ECM, and/or to contribute to the electrocyte’s electrical properties through interaction with voltage gated ion channels.

A second notable pattern in our results is the unusual degree to which mormyrid electrocytes retain expression of some sarcomeric genes, which has been noted in several studies [58, 65, 66, 75, 76]. The role these proteins serve in electrocytes is presently unknown; however, our results indicate that some are highly differentially expressed between *Parmormyrops* with different EOD waveforms (Tables 4-6). This strongly suggests that sarcomeric proteins could play an important role in the conformational changes required to develop and sustain penetrations.

Finally, the biochemical composition and function of the ECM in electrocytes is poorly understood. Our analysis identifies differential expression in ECM-related genes across the genus *Paramormyrops*, associated with each of the three EOD features studied. At least four of these genes (*cell migration-inducing and hyaluronan-binding protein-like, inter-alpha-trypsin inhibitor heavy chain H5-like* and two copies of *inter-alpha-trypsin inhibitor heavy chain 3*), distributed across two EOD features, interact with hyaluronan. Hyaluronan is a type of mucopolysaccharide and a major component of some soft tissues and fluids [77]. Therefore, we propose that hyaluronan is an important constituent of the ECM in mormyrid fish. In addition, the electrocyte-ECM interactions should be an important area of future investigation, as they are likely to influence electrocyte shape, electrical properties, and potentially the morphology of penetrations and surface membrane invaginations.

To conclude, this study examined the expression correlates of a hyper-variable phenotype in a rapidly diversified genus of mormyrid electric fish. We examined DGE between taxa exhibiting variability along three major axes of variation that characterize EOD differences within *Paramormyrops* and among mormyrids: duration, polarity, and complexity. We found that gene expression in EOs among closely related species is largely similar, but patterns of DGE between EOs is primarily restricted to four broad functional sets: (1) cytoskeletal and sarcomeric proteins, (2) cation homeostasis, (3) lipid metabolism and (4) proteins that interact with the ECM. Our results suggest specific candidate genes that are likely to influence the size, shape and architecture of electrocytes for future research on gene function and molecular pathways that underlie EOD variation in mormyrid electric fish.

## Methods

### Sample collection

We captured 11 *Paramormyrops* individuals from Gabon, West Central Africa in 2009: five *P. kingsleyae* (*n* = 3 N-type and *n* = 2 P-type), four *P.* sp. *‘*magnostipes’ (n = 2 Type I and n = 2 Type II), and two *P.* sp. ‘SN3’. Within 1–12 h of capture, individual specimens were euthanized by overdose with MS-222. The caudal peduncle was excised and skinned, and immediately immersed in RNA-later for 24 h at 4 °C, before being transferred to − 20 °C for long-term storage. As two of these species (*P.* sp. ‘magnostipes’, *P.* sp. ‘SN3’) are presently undescribed, we note that these specimens were identified by their EOD waveform, head morphology and collecting locality [30, 31, 35, 78]. All specimens, including vouchers materials, are deposited in the Cornell University Museum of Vertebrates. Collection information and the phenotypes per EOD feature of each sample are detailed in Table 1.

### RNA extraction, cDNA library preparation and Illumina sequencing

Total RNA was extracted from EOs using RNA-easy Kit (Qiagen, Inc) after homogenization with a bead-beater (Biospec, Inc.) in homogenization buffer. mRNA was isolated from total RNA using a NEBNext mRNA Isolation Kit (New England Biolabs, Inc.). Libraries for RNA-seq were prepared using the NEBNext mRNA Sample Prep Master Mix Set, following manufacturer’s instructions. Final libraries after size selection ranged from 250 to 367 bp. Libraries were pooled and sequenced by the Cornell University Biotechnology Resource Center Genomics Core on an Illumina HiSeq 2000 in a 2x100bp paired end format. Raw sequence reads were deposited in the NCBI SRA (Additional file 1).

### Read processing and data exploration

FastQC v0.11.3 (Babraham Bioinformatics) was used to manually inspect raw and processed reads. We used Trimmomatic v.0.32 [79] to remove library adaptors, low quality reads, and filter small reads; following the suggested settings of MacManes [80]: 2:30:10 SLIDINGWINDOW:4:5 LEADING:5 TRAILING:5 MINLEN:25. After trimming, reads from each specimen were aligned to the predicted transcripts of the NCBI-annotated (Release 100) *P. kingsleyae* (N-type) genome [58] using bowtie 2 v2.3.4.1 [81]. Expression quantification was estimated at the gene level using RSEM v1.3.0 [82], followed by exploration of the data with a gene expression correlation matrix based on Euclidean distances and Pearson’s correlation coefficient (for genes with read counts > 10, Trinity’s default parameters). All these steps were executed using scripts included with Trinity v2.6.6 [83, 84].

### Data analysis

We began by examining DGE between all possible pairwise comparisons of OTUs (*n* = 10, Table 2) using edgeR v3.20.9 [85] through a script provided with Trinity (MA plots are provided in Additional File 8). We restricted our consideration of genes to those where CPM-transformed counts were > 1 in at least two samples for each comparison (edgeR default parameters). We modified this to use the function estimateDisp() instead of the functions estimateCommonDisp() and estimateTagwiseDisp(). For each comparison, we conservatively considered genes to be differentially expressed with a minimum fold change of 4 and *p*-value of 0.001 after FDR correction. We compiled a non-redundant list of genes that were differentially expressed in at least one comparison based on these criteria (Fig. 3, Set A).

For each of the DEGs in Set A we used TMM normalized values to compare gene expression between groups of OTUs with alternative EOD waveform phenotypes (i.e. *long duration EOD* vs. *short duration EOD, biphasic* vs. *triphasic* and *small penetrations* vs. *large penetrations*, see Table 1. Note that waveform polarity phenotypes only apply to triphasic individuals). For each of the three phenotype pairs, we calculated the mean and standard deviation for TMM values within each grouping, then extracted the genes that had (1) expression values more than four times greater in one phenotype than the other and (2) a difference in mean expression greater than either within-group standard deviation. This resulted in six lists of upregulated genes, one for each EOD feature across all OTUs and samples (Fig. 3, Set B).

In order to assess enrichment of particular gene pathways, biological functions, and cellular locations using a controlled vocabulary, we performed GO [86, 87] enrichment tests on every list of upregulated genes from (1) the ten pairwise comparisons (*n* = 20, two per comparison), (2) Set B (*n* = 6), and (3) Set C (*n* = 6), for each of the three ontology domains: Biological Process, Cellular Component, and Molecular Function. First, we identified homologous proteins predicted from the *P. kingsleyae* (N-type) reference genome and those predicted from *Danio rerio* (GRCz11) by blastp (BLAST+ v2.6, [88]). For each protein, the top hit (e-value ≤1e-10) was used for annotation. Next, we used mygene v1.14.0 [89, 90] to match the *D. rerio* proteins to *D. rerio* genes and extract their GO annotations (zebrafish Zv9). This resulted in GO annotations for each of the three ontology domains for *P. kingsleyae* (N-type) genes. Finally, we carried out the GO enrichment tests using topGO v2.30.1 [91] and the following parameters: nodeSize = 10, statistic = fisher, algorithm = weight01, *p*-value ≤0.02. The ‘universe’ for each enrichment test on gene lists from the pairwise comparisons was all the genes deemed expressed in the respective comparison, whereas the non-redundant list of genes in these ten ‘universes’ was the ‘universe’ for all enrichment tests on the gene lists from Sets B and C.

Interpretation of lists of genes from Set A and Set B each suffered limitations for the overall goals of this analysis, which is to identify the DEGs most strongly associated with each waveform feature (duration, complexity, and polarity). The ten comparisons made to construct Set A were not equally informative for two primary reasons: (1) the OTUs in this analysis vary in terms of their phylogenetic relatedness (see [30, 31]) and (2) several OTU comparisons varied in more than one waveform characteristic (Table 2). As such, we elected to focus on the most informative comparison for each EOD feature: the comparison that contrasted only the given feature and that minimized phylogenetic distance between OTUs. Of the ten pairwise comparisons, we classified three as the most informative comparisons, one per EOD feature (Table 2). The six lists of significantly upregulated genes (fold change > 4, FDR-corrected p-value < 0.001) from these three pairwise OTU comparisons constitute Set A’.

Comparisons in Set A’, however, lack biological replication. In contrast, interpretations of Set B were potentially limited in that many of the OTUs in this analysis differed in more than one EOD feature. To circumvent the limitations of Sets A’ and B within the limits of our study design, we constructed a third set (Set C). Set C is defined as the intersection of the upregulated genes from Sets A’ and B, for each phenotype. Since there were six phenotypes in our study, Set C encompasses six lists of upregulated genes and their respective enriched GO terms (Fig. 3). Therefore, Set C represents the DEGs that are (1) statistically supported as differentially expressed between closely-related OTUs that vary in a single waveform characteristic, and (2) are consistently differentially expressed among all OTUs that share that waveform feature. We focus our attention on Set C: We retrieved GO term definitions from QuickGO [92] and descriptions of gene function of the functional annotations from UniProt [93]; and to facilitate the discussion, we classified the more interesting genes in Set C into “general” functional classes, or themes.

## Supplementary information

**Additional file 1 **Raw reads NCBI SRA accession numbers, number of reads and alignment rates per sample, using bowtie 2 as the aligner and the *Paramormyrops kingsleyae* (N-type) transcriptome as the reference.

**Additional file 2.** DEG per comparison from the 10 pairwise DGE analysis. Positive values under logFC indicate genes upregulated in the OTU under sampleA, whereas negative values correspond to genes upregulated in the OTU under sampleB. Values under each sample are gene raw counts. Significance threshold was abs (log (base2)FC) > 2 (= 4-fold expression difference) and FDR < 0.001.

**Additional file 3.** Enriched GO terms per comparison, ontology and OTU in the DEG from the 10 pairwise comparisons. Also listed are the DEG annotated to each GO term. The pvalue is in the column weight01.

**Additional file 4.** DEG per EOD feature and phenotype identified with the Set B analysis. Values under each sample are TMM normalized, log2(TMM + 1) transformed, and mean-centered expression values.

**Additional file 5.** Enriched GO terms per EOD feature, ontology and phenotype in the DEG from the Set B analysis. Also listed are the DEG annotated to each GO term. The pvalue is in the column weight01.

**Additional file 6.** DEG in Set C, per EOD feature and phenotype.

**Additional file 7.** GO terms enriched in the DEG in Set C, per EOD feature, ontology and phenotype. Also listed are the DEG annotated to each GO term, and the quickGO definitions of each GO term. The pvalue is in the column weight01.

**Additional file 8.** MA plots from the 10 pairwise DGE analysis. Red dots represent genes with FDR < 0.05 (Trinity’s default parameters).

## Data Availability

Raw sequence reads for all samples were deposited in the NCBI SRA with the BioProject Accession Number PRJNA573805. Per sample SRA accession numbers are listed in Additional file 1. All source code necessary to perform the methods described in this manuscript is provided in a GitHub repository: http://github.com/msuefishlab/paramormyrops_rnaseq

## Abbreviations

DEG: Differentially expressed gene
DGE: Differential gene expression
ECM: Extracellular matrix
EO: Electric organ
EOD: Electric organ discharge
GO: Gene Ontology
NCBI: National Center for Biotechnology Information
OTU: Operational taxonomic unit
