# Supplementary material for: The transcriptional correlates of divergent electric organ discharges in Paramormyrops electric fish
Source: BMC Evol Biol. 2020 Jan 9;20:6. doi: 10.1186/s12862-019-1572-3 (PMC6953315; doi:10.1186/s12862-019-1572-3)
Supplement: Supplementary file 8 — Additional file 8. MA plots from the 10 pairwise DGE analysis. Red dots represent genes with FDR < 0.05 (Trinity’s default parameters). [file 12862_2019_1572_MOESM8_ESM.pdf]

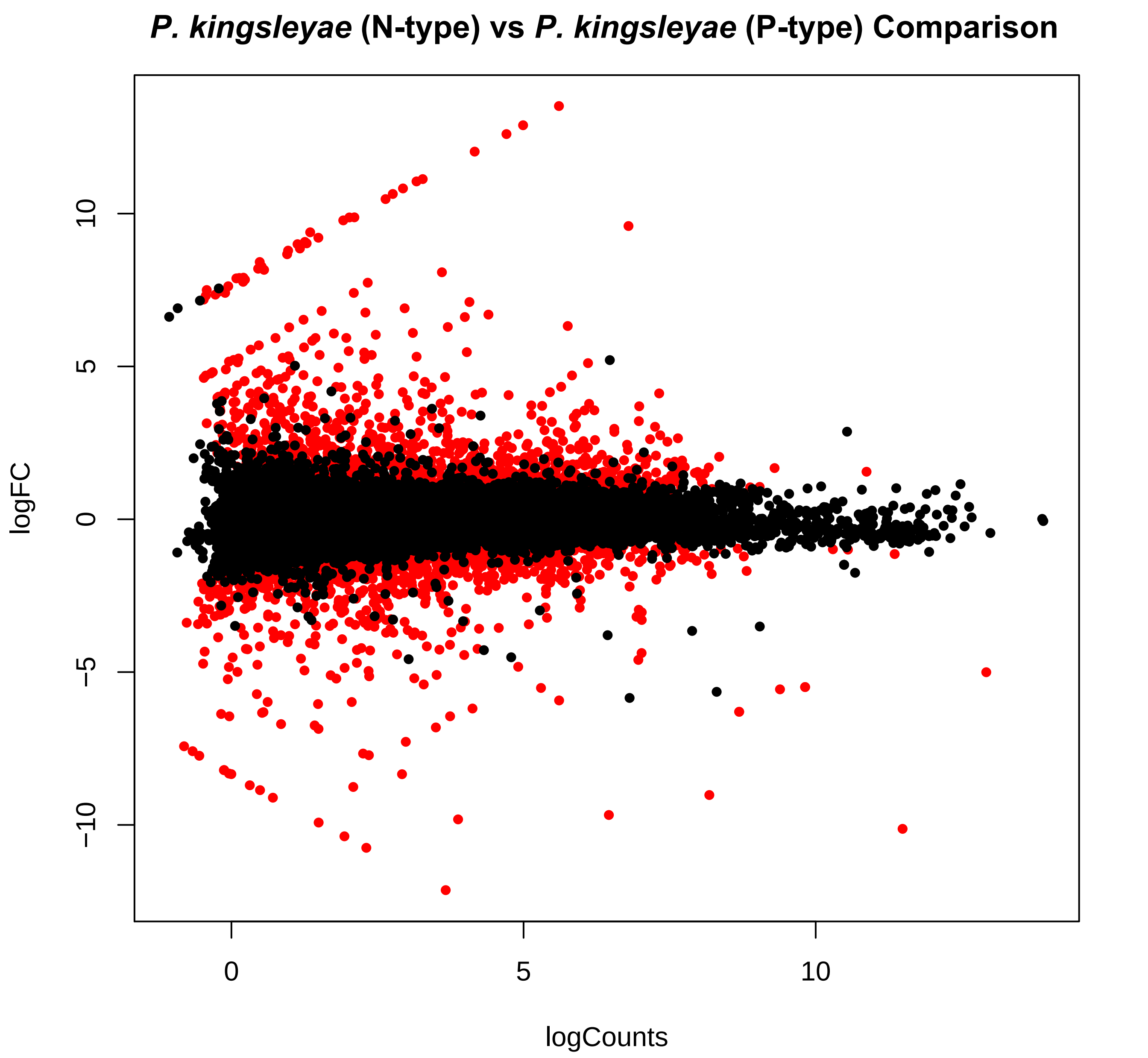
P. kingsleyae (N-type) vs *P. kingsleyae* (P-type) Comparison
logFC
10
5
0
−5
−10
logCounts
0
5
10

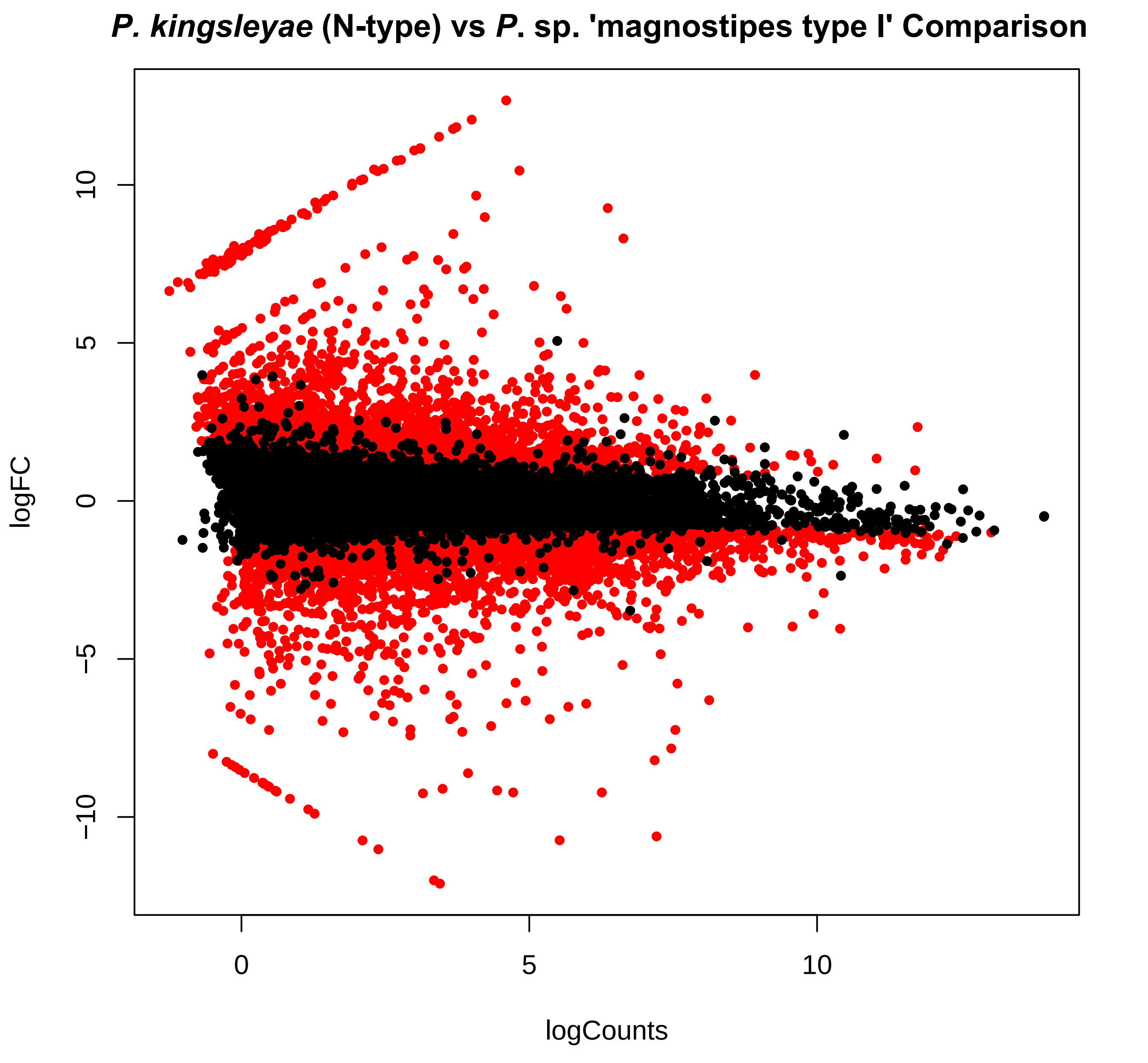

P. kingsleyae (N-type) vs P. sp. 'magnostipes type I' Comparison
logFC
logCounts
10
5
0
−5
−10
0
5
10

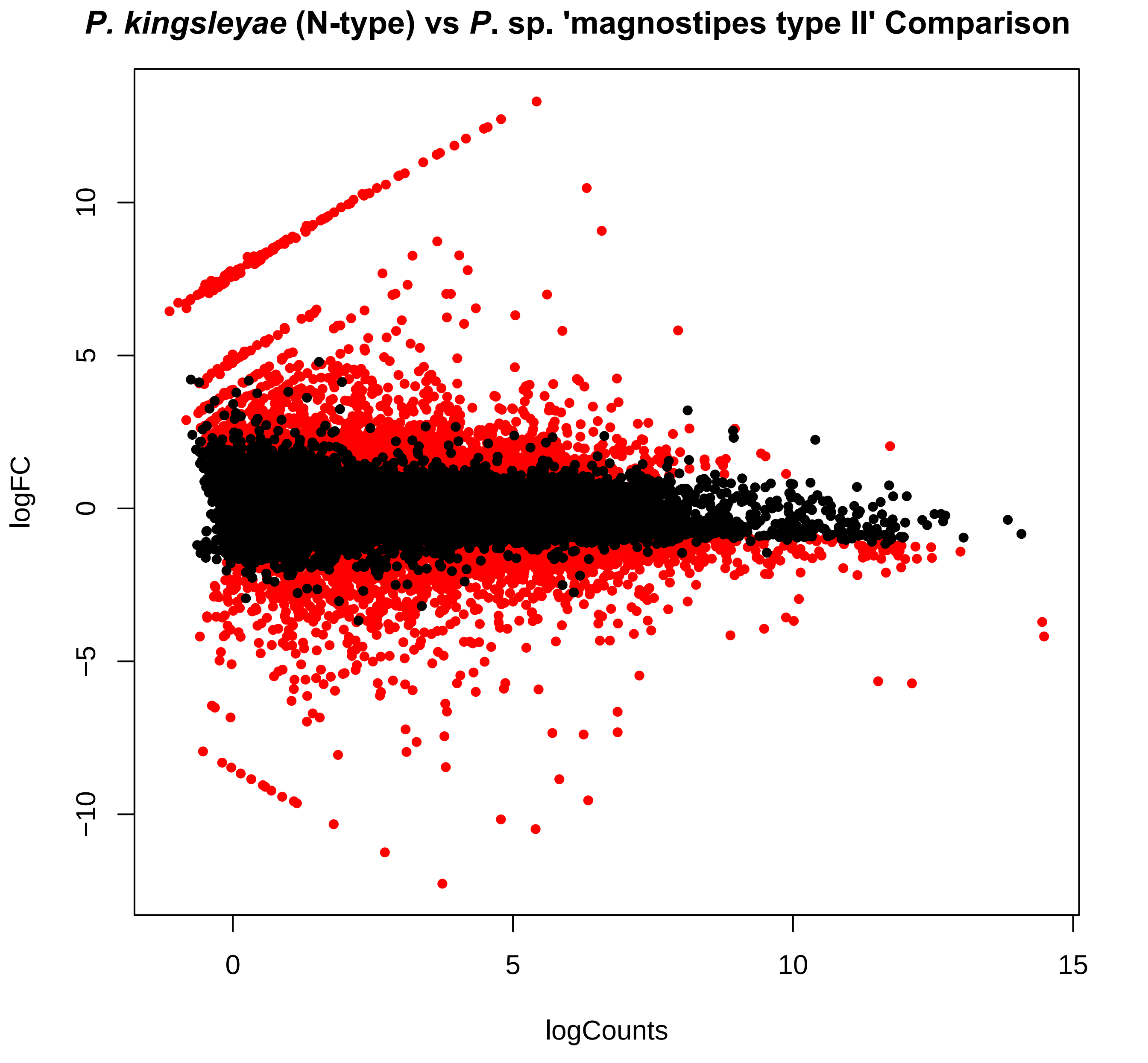
P. kingsleyae (N-type) vs *P.* sp. 'magnostipes type II' Comparison
logFC
logCounts
10
5
0
−5
−10
0
5
10
15

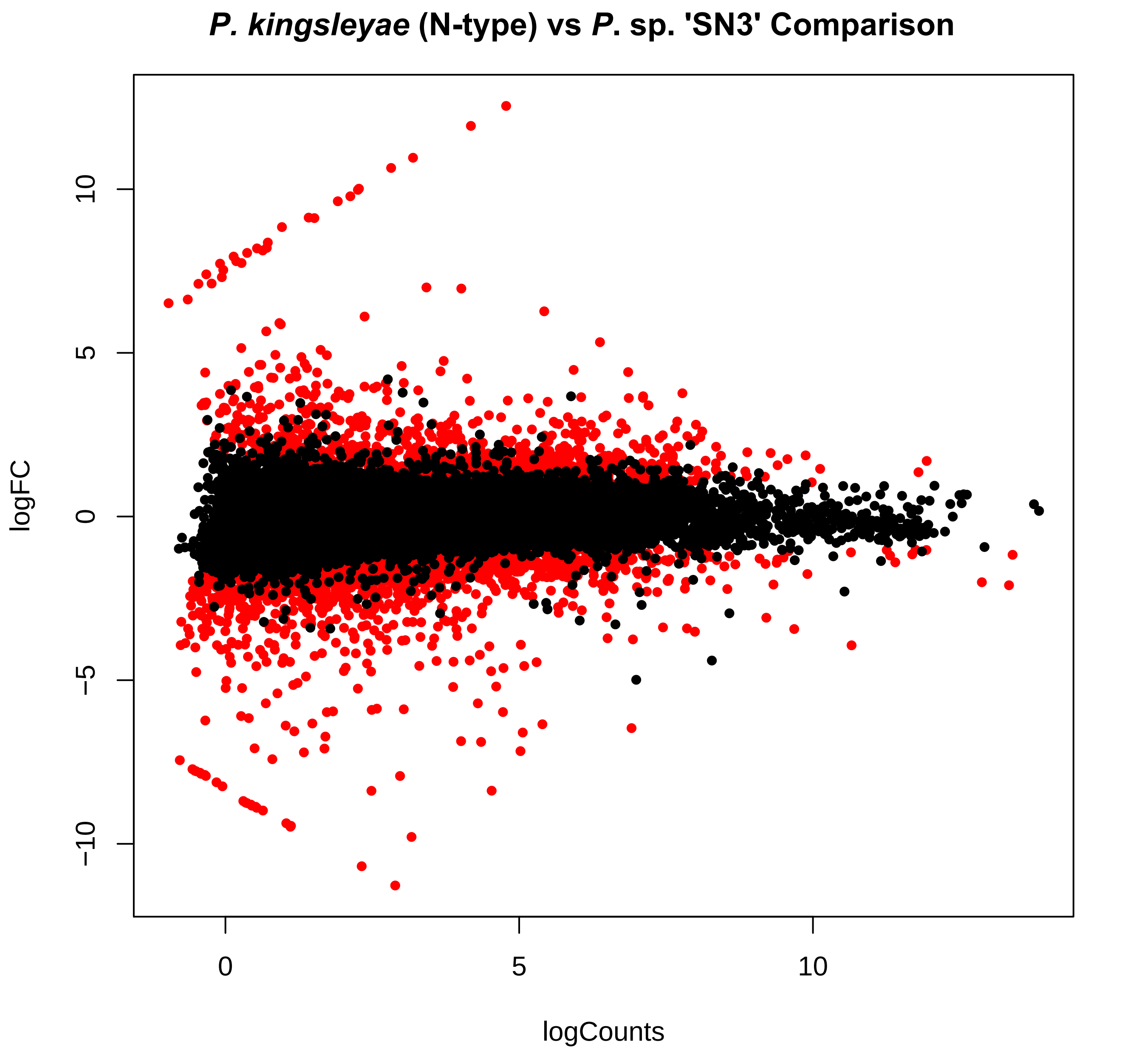
P. kingsleyae (N-type) vs *P*. sp. 'SN3' Comparison
logFC
10
5
0
−5
−10
0
5
10
logCounts

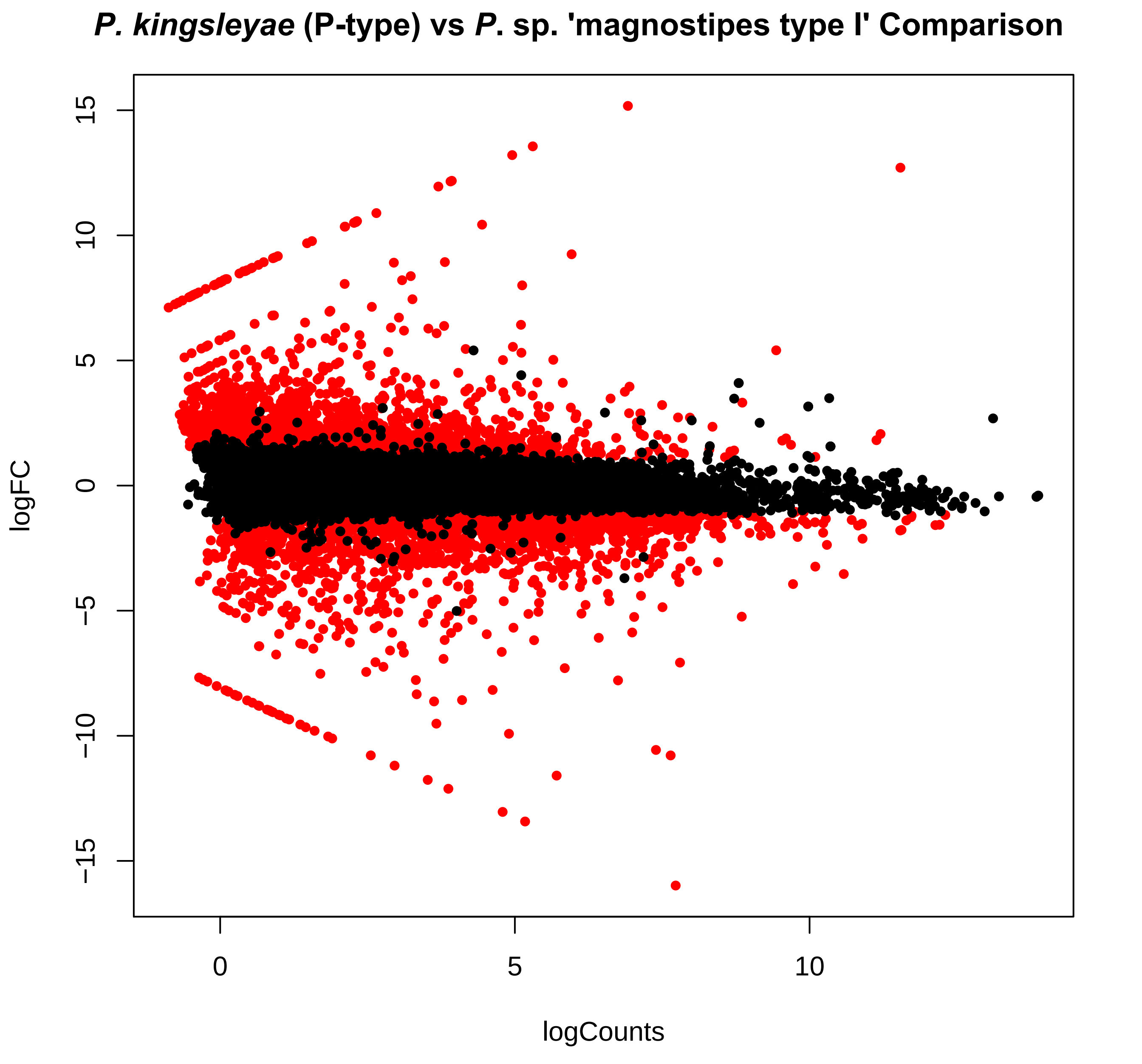

P. kingsleyae (P-type) vs *P*. sp. 'magnostipes type I' Comparison
logFC
logCounts
15
10
5
0
−5
−10
−15
0
5
10

P. kingsleyae (P-type) vs *P*. sp. 'magnostipes type II' Comparison

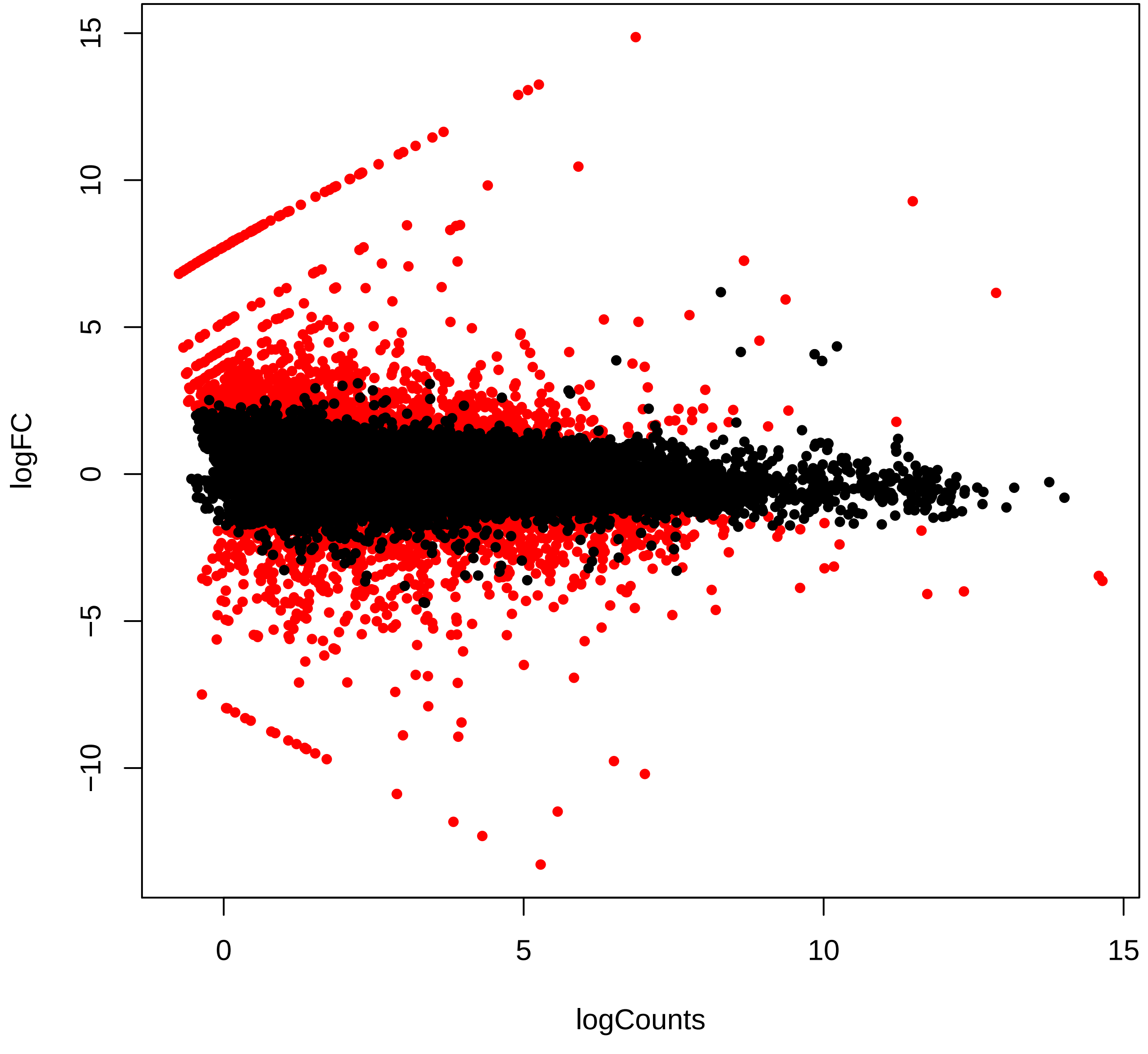

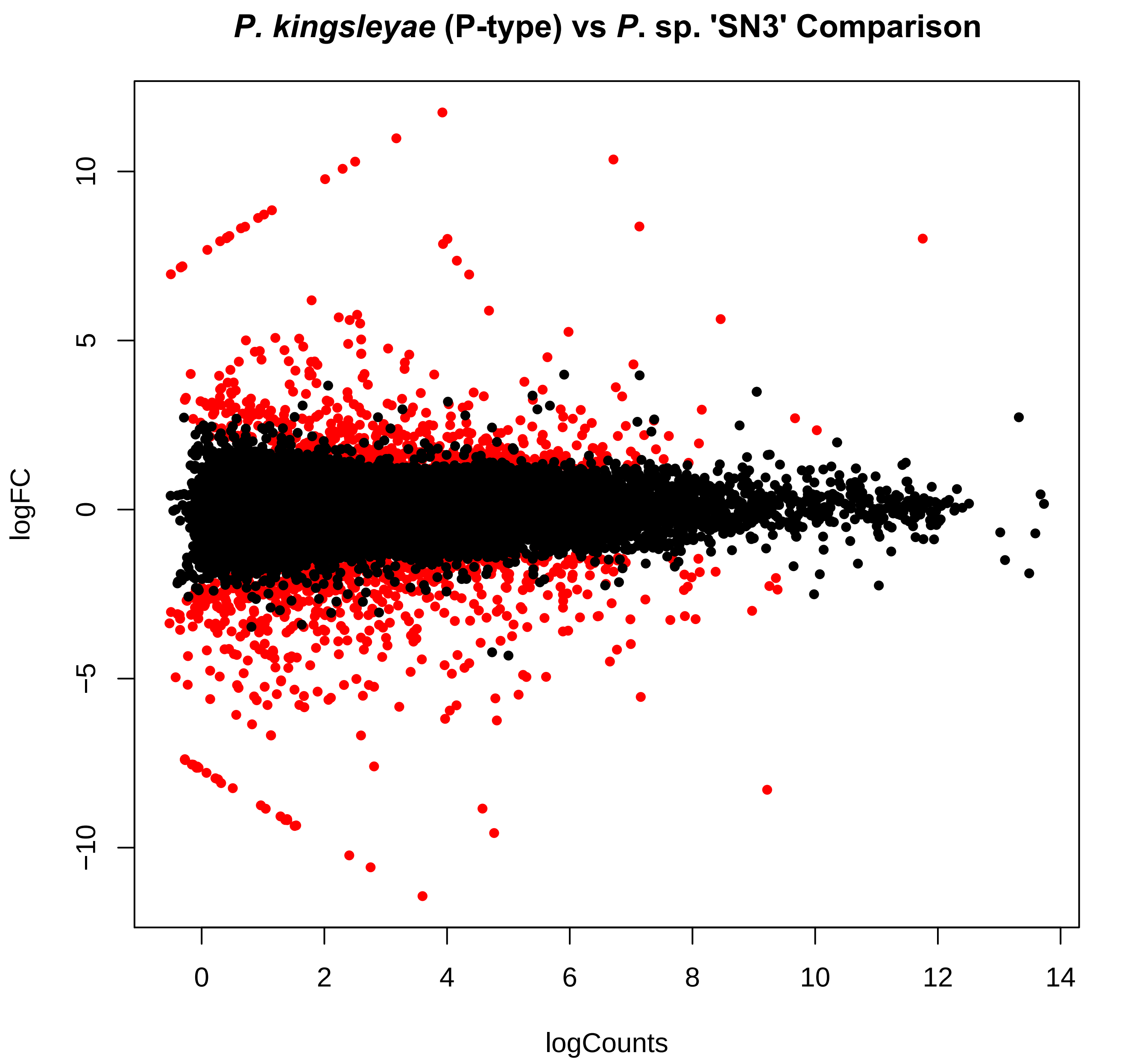

P. kingsleyae (P-type) vs *P.* sp. 'SN3' Comparison
logFC
logCounts
10
5
0
−5
−10
0
2
4
6
8
10
12
14

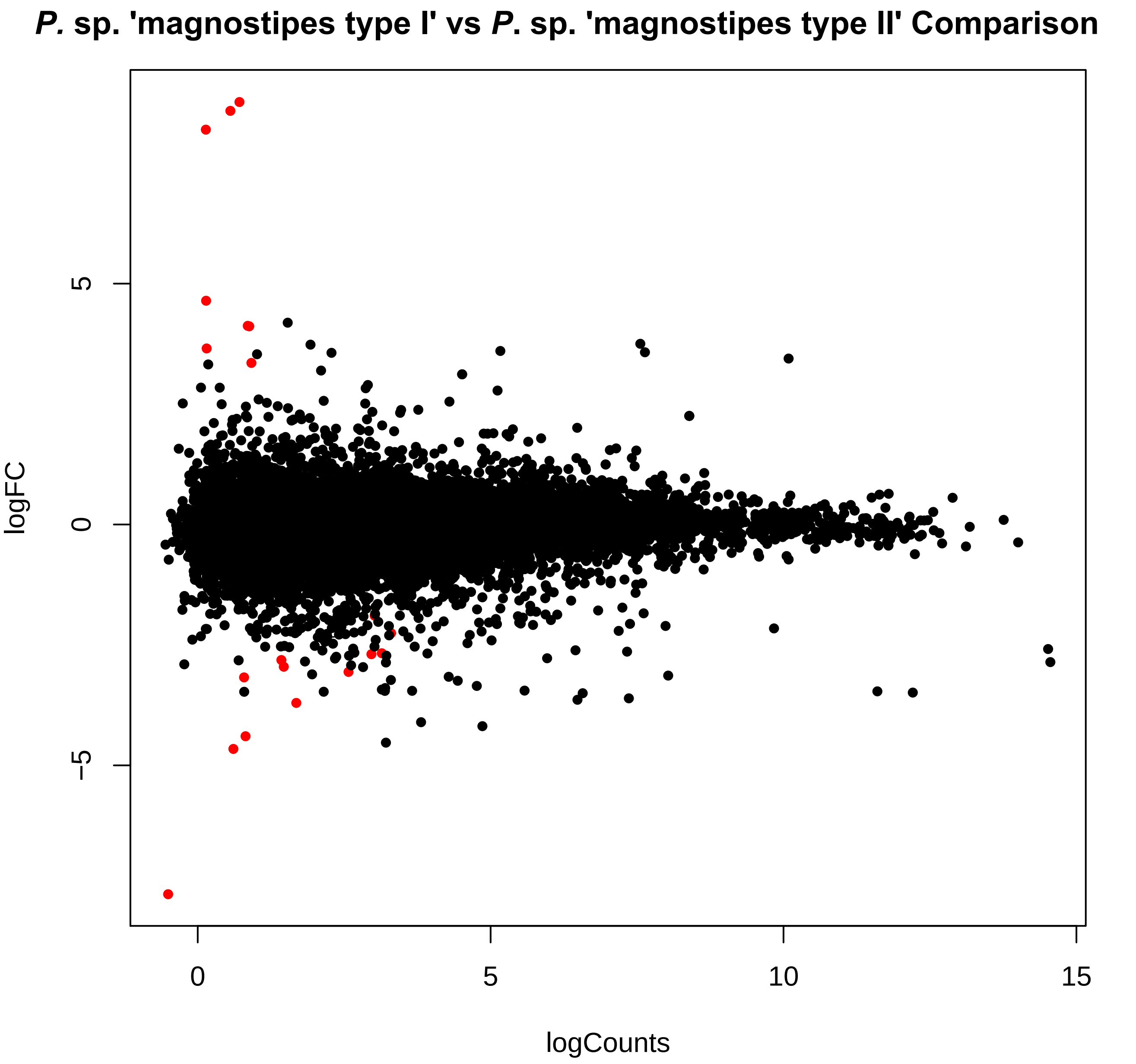

P. sp. 'magnostipes type I' vs *P.* sp. 'magnostipes type II' Comparison
logFC
5
0
−5
0
5
10
15
logCounts

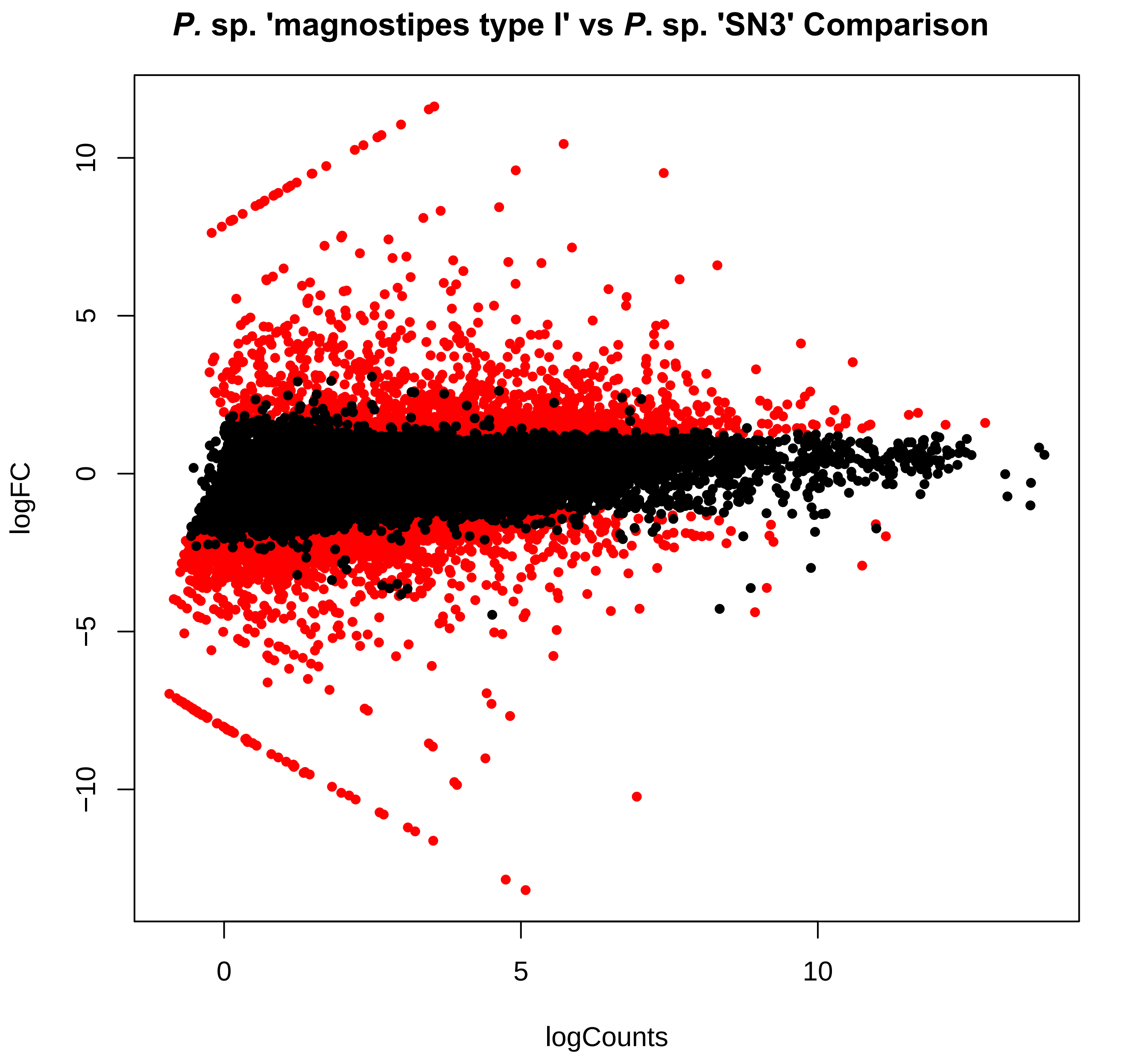
P. sp. 'magnostipes type I' vs *P*. sp. 'SN3' Comparison
logFC
logCounts
10
5
0
−5
−10
0
5
10

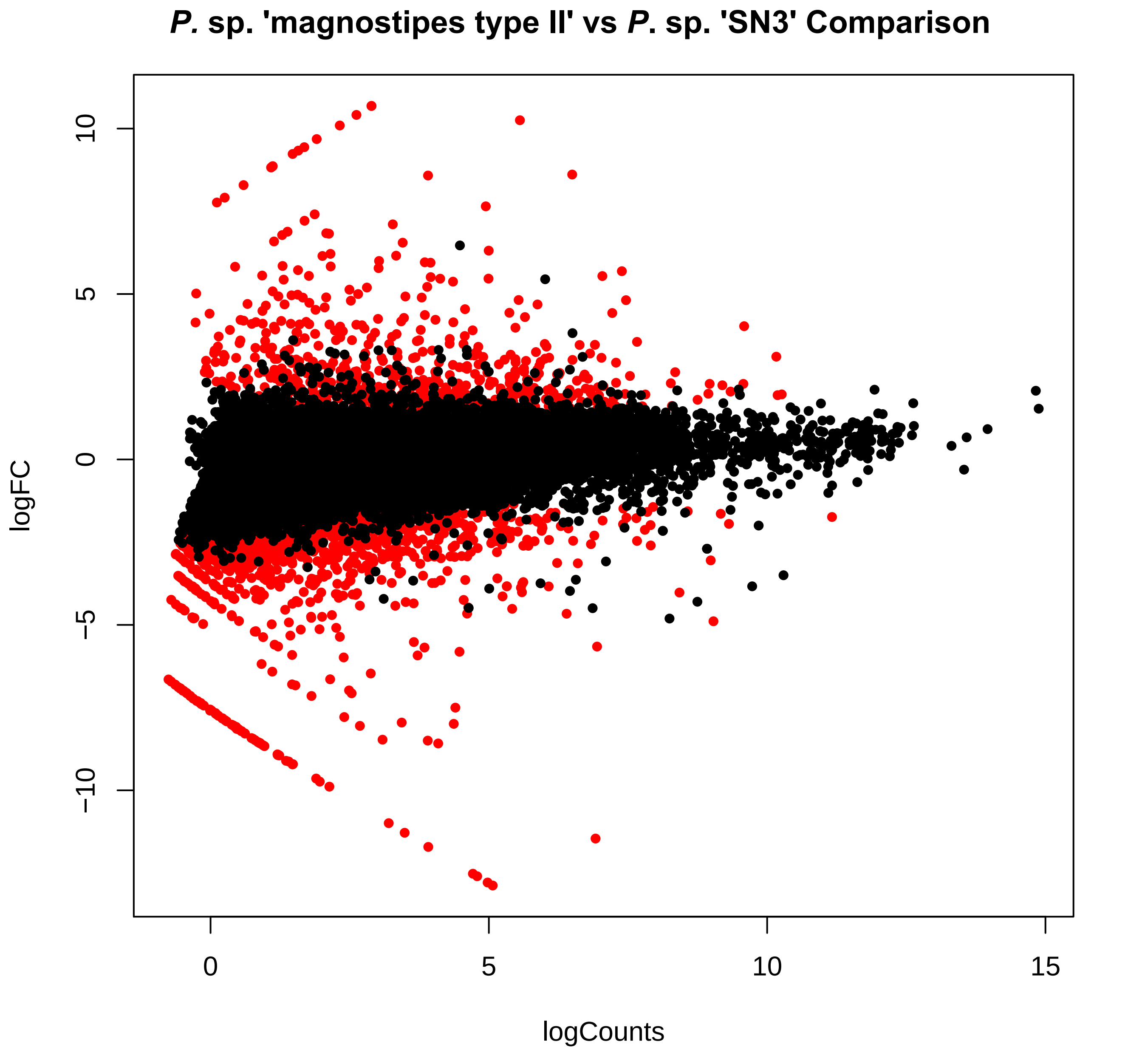
P. sp. 'magnostipes type II' vs *P*. sp. 'SN3' Comparison
logFC
logCounts
10
5
0
−5
−10
0
5
10
15
